# Trends and Correlates of High-Risk Alcohol Consumption and Types of Alcoholic Beverages in Middle-Aged Korean Adults: Results From the HEXA-G Study

**DOI:** 10.2188/jea.JE20170296

**Published:** 2019-04-05

**Authors:** Jaesung Choi, Ji-Yeob Choi, Aesun Shin, Sang-Ah Lee, Kyoung-Mu Lee, Juhwan Oh, Joo Yong Park, Jong-koo Lee, Daehee Kang

**Affiliations:** 1 Department of Biomedical Sciences, Seoul National University Graduate School, Seoul, Korea; 2 Department of Preventive Medicine, Seoul National University College of Medicine, Seoul, Korea; 3 Cancer Research Institute, Seoul National University, Seoul, Korea; 4 Department of Preventive Medicine, Kangwon National University School of Medicine, Kangwon, Korea; 5 Department of Environmental Health, College of Natural Science, Korea National Open University, Seoul, Korea; 6 JW Lee Center for Global Medicine, Seoul National University College of Medicine, Seoul, Korea; 7 Department of Family Medicine, Seoul National University College of Medicine, Seoul, Korea; 8 Institute of Environmental Medicine, Seoul National University Medical Research Center, Seoul, Korea

**Keywords:** alcohol consumption, prevalence, trends, correlates

## Abstract

**Background:**

We aimed to report the prevalence and correlates of high-risk alcohol consumption and types of alcoholic beverages.

**Methods:**

The baseline data of the Health Examinees-Gem (HEXA-G) study participants, including 43,927 men and 85,897 women enrolled from 2005 through 2013, were used for analysis. Joinpoint regression was performed to estimate trends in the age-standardized prevalence of alcohol consumption. Associations of demographic and behavioral factors, perceived health-related effects, social relationships, and the diagnostic history of diseases with alcohol consumption were assessed using multinomial logistic regression.

**Results:**

The prevalence of alcohol consumption remained higher in men during the study period and increased in women. The amount of alcohol consumed has increased in women, especially that from beer and makgeolli, a traditional Korean fermented rice wine. Older participants were less likely to be high-risk drinkers (men and women who drink more than 40 or 20 g/day of alcohol, respectively) and drink Soju, a distilled liquor, and beer, and more likely to drink makgeolli. Educational level was negatively associated with high-risk drinking. However, it was positively associated with the consumption of strong spirits and wine. Smoking was associated with high-risk drinking and the consumption of soju and strong spirits. Engaging in regular exercise and having stress were associated with drinking all types of beverages except for soju.

**Conclusions:**

Sex-specific trends in alcohol consumption were influenced by demographic, behavioral, and perceived health-related factors. The findings will help improve the understanding of alcohol-related problems and provide evidence for establishing country-specific policies and campaigns in Korea.

## INTRODUCTION

Alcohol consumption is one of the major risk factors for death and disease worldwide.^1^^,^^2^ Korea is among the countries with the highest alcohol consumption in the world, and alcohol is the second leading cause of disability-adjusted life years (DALY).^2^ Since Koreans consume not only internationally popular alcoholic beverages, such as beer, whiskey, and wine, but also traditional alcoholic beverages, how various alcoholic beverages influence total alcohol consumption should be evaluated.

The impact of alcohol consumption on health varies depending on the amount of alcohol and types of alcoholic beverages consumed. The World Health Organization (WHO) has suggested that alcohol drinking has an impact on the chronic harm it causes according to the amount of alcohol consumed.^3^ Several studies have confirmed that heavy drinking is known to cause various illnesses or trauma.^1^^,^^4^^–^^7^ The types of alcoholic beverages consumed are also known to be associated with mortality or risk of diseases. For example, it has been suggested that the consumption of beer or strong spirits in Western countries has more harmful effects on mortality, CVD, or cancer than wine.^8^^–^^10^

Previous studies evaluated the prevalence or correlates of alcohol consumption depending on distinctive alcohol-related cultures.^11^^–^^18^ Younger age, higher socioeconomic status (SES), and smoking were consistently found to have positive associations with alcohol consumption in various countries.^15^^–^^18^ It was reported that beer drinkers are more likely to be high-risk drinkers compared to wine drinkers,^19^ while consumers of strong spirits are more likely to be high-risk drinkers compared to beer drinkers.^20^ In Korea, it was reported that the trends in high-risk drinking fluctuated between 2008 and 2014 in men but not in women,^21^ and associations were observed between a younger age and a lower educational level and the amount of alcohol consumption.^22^^,^^23^ However, the prevalence and correlates by types of alcoholic beverages have not been assessed.

Therefore, we evaluated the trends in age-standardized prevalence and correlates of high-risk consumption of alcohol according to WHO guidelines^3^ and the types of alcoholic beverages.

## MATERIALS AND METHODS

### Study population

The baseline information of participants in the Health Examinees-Gem (HEXA-G) study, who were derived from the Health Examinees study, a component of the Korean Genome and Epidemiology Study (KoGES_HEXA), which recruited participants aged 40–69 years at 38 general hospitals and health examination centers in eight regions from 2004 through 2013, was used for analysis. The design of the KoGES_HEXA cohort study has been described elsewhere.^24^^,^^25^ Well-trained interviewers used a structured questionnaire to collect information on socio-demographic characteristics, medical history, medication usage, family history, and lifestyle factors, including smoking habits, alcohol consumption habits, weight control, and regular exercise participation. Information on reproductive history was also collected from the women, and skilled medical staff performed a physical examination on all the participants. During recruitment, all the participants voluntarily signed a consent form before entering the study, and the Institutional Review Board (IRB) of Seoul National University Hospital, Seoul, Korea approved the study (IRB No. 0608-018-179). In the HEXA-G study, 139,348 participants, comprising 46,978 men (33.7%) and 92,370 (66.3%) women, were included at baseline, after excluding participants recruited at 21 centers (*n* = 30,374). Exclusion criteria for the recruiting center were described in a previous study.^26^ Briefly, sites that only operated in the pilot study years, that have different processes for quality control and biospecimen collection, and that have been participating for fewer than 2 years were excluded. Among the HEXA-G participants, those who did not provide information on alcohol consumption status or duration of alcohol cessation were excluded (*n* = 9,524). Those missing data on any of the other variables were not excluded. Finally, 129,824 participants, including 43,927 men (33.8%) and 85,897 women (66.2%), were included in the analyses.

### Alcohol consumption

Alcohol consumption was assessed by asking whether the participants drank alcohol. Never drinkers were defined as those who had never drank alcohol in their lives for any reasons; former drinkers were those who had not drank alcohol in the prior 12 months, but had consumed it in the past; and current drinkers were those who had drunk alcohol in the prior 12 months. Because the former drinkers were more likely to have a diagnosis of disease (eTable 1 and eTable 2), only the results for the current drinkers were presented in the main analysis. Among the current drinkers, information on the frequency of alcohol consumption per month, week, and day and the number of drinks each time were collected according to the type of alcoholic beverage, such as soju (the most widely consumed distilled liquor in Korea), beer, makgeolli (a traditional fermented rice wine), strong spirits, wine, and cheongju (a distilled rice liquor).^20^ Those who reported drinking each type of alcoholic beverage at least once in the prior year were regarded as drinkers of the alcoholic beverage, whereas those who drank alcohol but did not drink a certain type of alcoholic beverage were categorized as drinkers of other types of beverages. If there was no information on the frequency of alcohol consumption, the status of drinking each type of alcoholic beverage was categorized as missing. The participants were not exclusively categorized as drinkers of each type of beverage because some drank more than one type of alcohol. For example, a participant who drank beer and wine was counted twice, once as a beer drinker and once as a wine drinker. The total amount of alcohol consumed per day was assessed by summing the alcohol consumed per day from all types of alcoholic beverage after multiplying the number of drinks each time, the average frequency per day, and the standard ethanol content of one drink (soju: 19%; beer: 5%; makgeolli: 6%; strong spirits: 43%; wine: 13%; and cheongju: 13%).^27^ The amount of alcohol consumption was categorized as low-, medium-, and high-risk according to the criteria based on WHO guidelines for monitoring alcohol consumption (40 and 60 g per day for men and 20 and 40 g per day for women for medium- and high-risk, respectively).^3^

### Potential correlates

Demographic, behavioral, and perceived health-related factors, social relationships, and the diagnoses of diseases known to be associated with alcohol consumption a priori were selected as potential correlates and grouped into categories.^15^^–^^18^^,^^22^^,^^23^^,^^28^^,^^29^ The demographic factors included the following: age (40–44, 45–49, 50–54, 55–59, 60–64, and 65–69 years), education (≤middle school, high school, and ≥college), household income in Korean currency (<2 million won, 2–3.9 million won, and ≥4 million won), marital status (living with spouse or living alone), and current occupation (office, manual, unemployed, or housewife). The behavioral factors included the following: smoking status (never, former, and current), body mass index (BMI; <18.5, 18.5–24.9, 25.0–29.9, and ≥30 kg/m^2^) as an indicator of weight control, and duration of regular exercise (none, <150, and ≥150 min/week). The perceived health-related factors included the following: self-rated health (good, normal, and poor) and perceived stress in the prior month (not at all, often, and frequent). Other information was also collected: social relationships including contact frequency with family (none, <8, and ≥8 times per month) and close friends (none, <4, and ≥4 times per month); and diagnoses of the following diseases (no and yes): diabetes, myocardial infarction, stroke, cancer, acute liver disease, fatty liver, and cirrhosis.

### Statistical analysis

All of the analyses were processed separately by sex. An age distribution of the Korean mid-year population in 2005 (in 5-year groups) was used as a standard population to estimate the age-standardized prevalence. Joinpoint regression was used to estimate the annual percentage change (APC) of the age-standardized prevalence of never, former, and current drinkers and risk level of alcohol consumption in the total population.^30^ In current drinkers, the trends in the age-standardized mean of the total amount of alcohol consumption (g/day) and the percentage of alcohol brought by each type of alcoholic beverage were evaluated.

Odds ratios (ORs) and 95% confidence intervals (CIs) for association of each potential correlate with alcohol consumption or low-, medium-, and high-risk drinking were evaluated using a multinomial logistic regression model compared to never drinkers. To avoid multicollinearity, we checked the variance inflation factors (VIF) for potential correlates that were associated with alcohol consumption (eTable 1 and eTable 2) in men or women and excluded the variables with VIF greater than 10 from the model. Finally, all potential variables were included in the model. Differences between the groups according to low- and high-risk were estimated by testing linear hypotheses about the regression coefficients.^31^ Because the association of potential correlates with medium- and high-risk consumption were not different (eTable 3 and eTable 4), those two categories were combined as high-risk. In the analysis by types of alcoholic beverage, the association of each potential correlate with consumption of soju, beer, makgeolli, strong spirits, and wine were compared to the consumption of other types of beverages using a logistic regression model because the drinkers of each type of alcoholic beverage were not exclusively categorized. The same model previously described was used. Because the prevalence of consuming cheongju was less than 5% in both men and women, the results for cheongju are not shown.

The JoinPoint Regression Program, version 4.4.0 (National Cancer Institute, Rockville, MD, USA), was used to conduct the joinpoint regression. SAS, version 9.4 (SAS Inc., Cary, NC, USA), was used to conduct the multinomial and multivariate logistic regression. All the tests were two-sided. Because the JoinPoint Regression Program only provided whether the *P* value was less than 0.05, the *P* value was considered an indicator of statistical significance in the joinpoint regression.

## RESULTS

### Prevalence and trends in alcohol consumption

**Figure 1.**
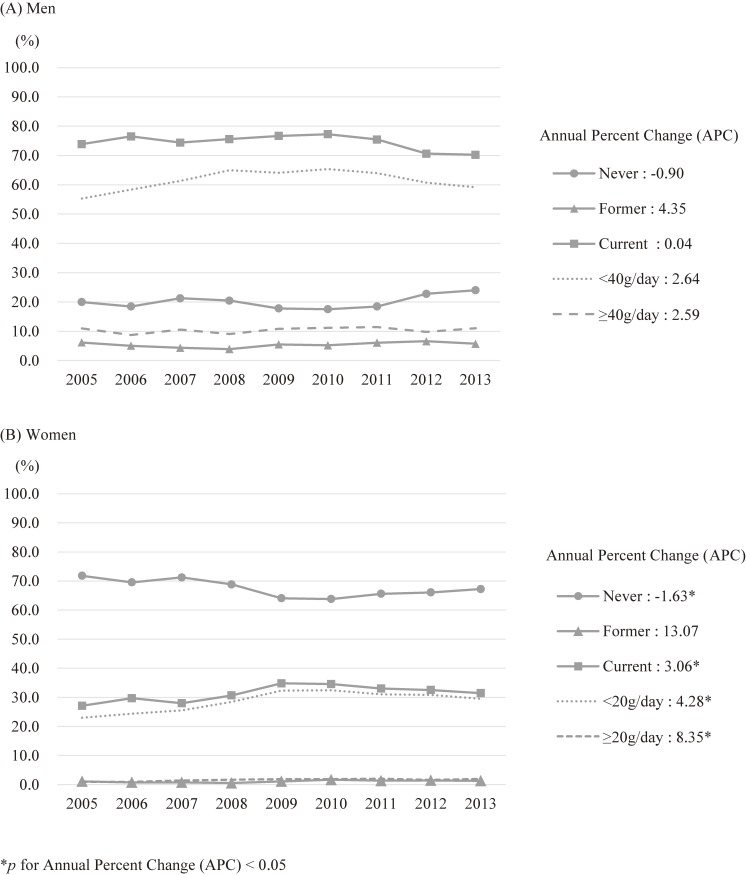
Trend of age-standardized prevalence of alcohol consumption among 43,927 men and 85,897 women aged 40–69 years in HEXA-G study from 2005 through 2013

**Figure 2.**
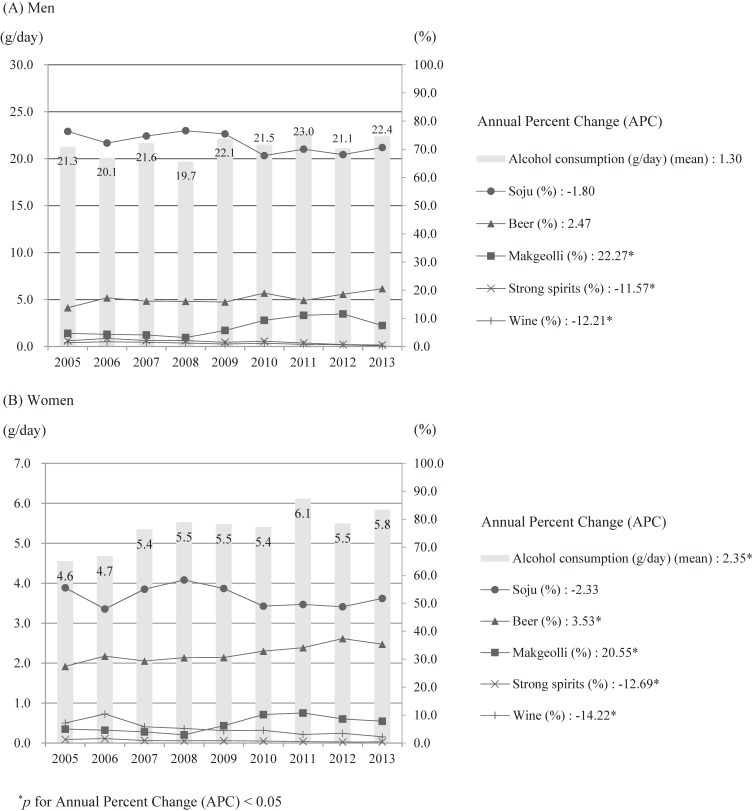
Trends in age-standardized mean of total alcohol consumption and percentage of alcohol brought by types of alcoholic beverage among 58,977 current drinkers (32,475 men and 26,502 women) aged 40–69 years in the HEXA-G study from 2005 through 2013

The mean and standard deviation (SD) of age in the study population was 53.7 (SD, 8.4) for the men and 52.4 (SD, 7.8) for the women. The age-standardized prevalence of current alcohol consumption was 75.7% in the men and 31.9% in the women. The trends in the age-standardized prevalence of alcohol consumption and low- or high-risk alcohol consumption appear in Figure 1. The prevalence of alcohol consumption did not change in the men. In women, the prevalence of current drinkers (27.1% in 2005 and 31.5% in 2013, APC = 3.06, *P* < 0.05) and both low- (23.0% in 2005 and 29.5% in 2013, APC = 4.28, *P* < 0.05) and high-risk drinkers (0.9% in 2005 and 1.9% in 2013, APC = 8.35, *P* < 0.05) increased. In the current drinkers, the mean age-standardized amount of alcohol consumption was 21.4 g/day in the men and 5.5 g/day in the women during the study period. There was a trend in the increase in the amount of alcohol consumption only in the women (4.6 g in 2005 and 5.8 g in 2013, APC = 2.35, *P* < 0.05), in whom the percentage of alcohol from beer (27.4% in 2005 and 35.4% in 2013, APC = 3.53, *P* < 0.05) and makgeolli increased (5.0% in 2005 and 7.8% in 2013, APC = 20.55, *P* < 0.05), while strong spirits (1.3% in 2005 and 0.4% in 2013, APC = −12.69, *P* < 0.05) and wine decreased (7.2% in 2005 and 2.2% in 2013, APC = −14.22, *P* < 0.05) (Figure 2). Among the five types of alcoholic beverages, soju was the most frequently consumed (Table 1). Those who drank strong spirits were most likely to be high-risk drinkers among both the men and women (25.6% in the men and 18.6% in the women).

**Table 1.** Frequency of consuming each type of alcoholic beverage and total alcohol consumption according to the types of alcoholic beverages consumed by 58,977 current drinkers (32,475 men and 26,502 women) aged 40–69 in the HEXA-G study

	Soju	Beer	Makgeolli	Strong spirits	Wine
Men					
Total,^a^ *N*	29,817	14,023	6,256	2,518	1,745
Frequency of alcohol consumption, %					
<1 time/week	27.1	43.5	52.2	79.9	73.4
1 time/week	22.8	26.0	20.3	10.5	12.3
2–3 times/week	35.3	23.9	17.5	7.1	9.6
≥4 times/week	14.8	6.6	10.0	2.4	4.8
Amount of alcohol consumption^b^, g/day, lsmean^c^	19.4	22.4	28.5	33.2	22.2
Risky amount of alcohol according to WHO guideline, %					
<40 g/day	83.4	80.6	72.6	67.8	80.1
≥40 g/day	14.1	16.2	22.9	25.6	13.2
Women					
Total,^a^ *N*	18,824	14,711	3,925	748	2,906
Frequency of alcohol consumption, (%)					
<1 time/week	65.2	66.8	73.3	79.1	77.5
1 time/week	19.1	18.4	15.9	9.6	11.4
2–3 times/week	13.1	11.9	8.3	7.8	8.2
≥4 times/week	2.6	3.0	2.4	3.5	2.9
Amount of alcohol consumption^b^, g/day, lsmean^c^	7.8	8.7	10.7	16.4	7.7
Risky amount of alcohol according to WHO guideline, %					
<20 g/day	91.5	90.8	87.3	73.4	89.6
≥20 g/day	5.9	6.5	8.5	18.6	3.9

WHO, World Health Organization.

Results of cheongju were not shown because of low prevalence (<5% in men and women). The total percentage of each category does not equal 100% because there were missing data.

^a^Drinkers of each alcoholic beverage were not exclusively categorized.

^b^Total amount of alcohol consumption among those who drink each type of alcoholic beverage.

^c^Least squares means (lsmean) were adjusted for age, education, household income, current occupation, marital status, smoking, body mass index, duration of regular exercise, self-rated health, stress, and contact frequency with family and close friends, and diagnosis history of diabetes, myocardial infarction, stroke, acute liver disease, fatty liver, and cirrhosis.

### Correlates of high-risk consumption of alcohol and the types of alcoholic beverages

**Figure 3.**
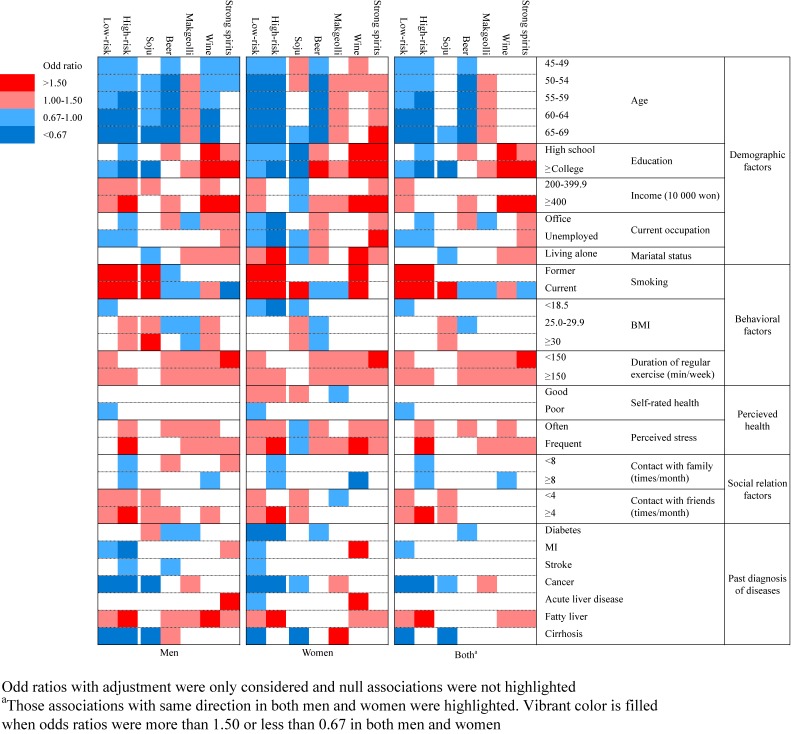
Summary of associations of potential correlates with patterns of alcohol consumption including risk levels and types of alcoholic beverages

Figure 3 presents the summary of the associations of potential correlates with low- or high-risk alcohol consumption and each type of alcoholic beverage in the men, women, and both (detailed ORs and 95% CIs are presented in eTable 5, eTable 6, eTable 7, eTable 8, eTable 9, and eTable 10). Among the demographic factors, the older participants were less likely to be high-risk drinkers in both the men and women. They preferred to drink makgeolli, whereas preference for soju or beer was lower than that of the younger participants. A higher educational level was negatively associated with high-risk drinking and soju drinking, although it was positively associated with other beverages. Office job employees and the unemployed were also negatively associated with high-risk drinking and positively associated with wine consumption. In the men, a higher level of household income was positively associated with high-risk drinking and the consumption of beer, strong spirits, and wine. In the women, living alone was positively associated with high-risk drinking and the consumption of beer, strong spirits, and wine.

Among the behavioral factors, smoking and regular exercise were positively associated with high-risk drinking in both the men and women. Smoking, the most strongly associated factor, was associated with the consumption of soju and strong spirits. Regularly exercising more than 150 minutes per week and having frequent stress were associated with all types of beverages except soju. A BMI higher than 25 was associated with high-risk drinking and the consumption of soju and strong spirits in the men.

Among the perceived health-related factors, frequent stress was positively associated with high-risk drinking and the consumption of makgeolli, strong spirits, and wine in both the men and women. The women who rated their health as “good” were more likely to be high-risk drinkers and consumers of soju.

Among the social relationship factors, frequent contact with family was negatively associated with high-risk drinking and the consumption of strong spirits; however, frequent contact with friends was positively associated with high-risk drinking and the consumption of soju.

## DISCUSSION

Sex-specific trends in alcohol consumption were influenced by demographic, behavioral, and perceived health-related factors. Soju was the most frequently consumed alcoholic beverage. Those who drank strong spirits were more likely to be high-risk drinkers. There were different associations between correlates and alcohol consumption according to the types of alcohol beverages. The present study further identified how each correlates relate to the consumption of various alcoholic beverages in Korea and the alcoholic beverages that may influence the trends in alcohol consumption in the Korean population.

The findings for the trends in the prevalence of alcohol consumption in our study were concordant with those of the Korean National Health and Nutrition Examination Survey (KNHANES), a nationally representative survey of participants aged 15 years or older. In the KNHANES, the age-standardized prevalence of alcohol consumption remained at a high level, from 72.6% to 75.2% in men, and increased from 36.9% to 46.5% in women between 2005 and 2015.^32^ In terms of the amount of alcohol, Korea is one of the countries with the highest alcohol consumption by men aged 15 years or older; they consume 45.6 g/day of pure alcohol, whereas women drink 13.0 g/day of pure alcohol according to a report by the WHO.^1^ However, those amounts were higher than those in our study population aged 40–69 years (21.4 g/day in the men and 5.5 g/day in the women). This difference could result from the presence of young participants aged 15 to 39 years, who had a higher prevalence of high-risk drinking, and from unrecorded alcohol consumption from sources such as homemade or informally produced alcohol, which added 20% to the WHO’s report.^1^ In our study, 91.8% of the male drinkers consumed soju and 71.0% of the women mainly consumed beer or spirits, unlike in many Western countries.^19^^,^^33^ The WHO reported that 70.5% of the alcohol consumption in the 2010 Korean population came from the “other beverage” category, which is comparable to our results showing the consumption of soju and makgeolli at 77.5% by men and 57.6% by women.^1^

Similar demographic factors, that those who were younger were more likely to be drinkers or high-risk drinkers, were consistently reported in previous studies.^17^^,^^22^^,^^23^^,^^34^ We found that beer and makgeolli were inversely associated with age, which was also reported in China, where beer consumption was higher in the younger generation, and consumption of a Chinese traditional alcoholic beverage, baijiu, was higher in older people.^17^ Higher education was negatively associated with high-risk drinking in both men and women, and higher levels of household income were positively associated with high-risk drinking in men. This finding reflects that educational inequalities have stronger effects on health behaviors than income levels in developed countries.^23^^,^^34^ The greater consumption of strong spirits and wine in those with higher education or household incomes may reflect the higher price of strong spirits and wine compared to other beverages in Korea.

Among the behavioral factors, consumption of beverages with higher alcohol content than other beverages, such as soju and strong spirits, was associated with smoking, which has been established as the most influential factor in high-risk drinking.^14^^–^^18^^,^^22^^,^^34^ The association of regular exercise with high-risk alcohol consumption was found in several previous cross-sectional studies,^18^^,^^22^^,^^35^^,^^36^ whereas null associations were found in longitudinal studies.^37^^–^^39^

Among the perceived health-related factors, those who reported their health as “good” were more likely to be high-risk drinkers than those who reported their health as “poor” in a previous study of 230,715 Koreans.^22^ Because previous prospective studies have shown that alcohol consumption does not affect the change in self-rated health,^40^^,^^41^ these results may reflect that those who think their own health is “good” tend to be inattentive to their health or overestimated their own health. Association of stress with alcohol consumption was consistently reported in a previous Korean study^22^^,^^34^ and in a meta-analysis of 11 European studies.^42^ These studies suggested that people drink alcohol to relieve stress, although alcohol consumption is a poor strategy for coping with stress, because alcohol itself may increase stress levels.^43^ Programs to educate people on coping with stress should be promoted.

Among the social relationship factors, our results for contact with family or friends reflected the evidence reported in previous studies, which reported the protective effects of family on high-risk alcohol consumption and the increased possibility of alcohol consumption when friends around the person were drinkers.^23^^,^^44^ Bosary et al suggested that the quality of relationships with family, friends, or significant others has a central role in substance use behaviors.^45^

There are many policies in Korea to reduce alcohol consumption, such as the regulation of advertising, mandatory warnings on alcoholic beverages, juvenile protection, and drunken driving control, but they have not been effective.^46^ The results of the present study suggest that it may be more efficient to promote policies or campaigns while considering correlates of high-risk alcohol consumption and specific alcoholic beverages.

This study had limitations that should be considered when interpreting its results. First, all of the variables were collected using a self-reported questionnaire. This can cause the underreporting of alcohol consumption or other unhealthy behaviors because of social desirability bias and recall bias. Second, only information on the six main types of alcoholic beverages was covered because information on other types of beverages was available only for a few recruiting years or there was no standard volume for those beverages. However, the results were expected to be unchanged because of the total frequency of those who reported drinking other types of alcoholic beverages was approximately less than 3% among the current drinkers. Third, the total frequency of those who drank two or more types of alcoholic beverages could not be assessed because the frequencies were collected by types of alcoholic beverages; we could not distinguish those who drank two or more types of beverages at once from those who drank them separately.

To conclude, this study found that there were sex-specific trends in alcohol consumption and increased percentages of alcohol from certain types of alcoholic beverages. Because there were inverse associations of age with beer and makgeolli, age-specific interventions should be performed to reduce these trends. Appropriate programs for at-risk populations, such as those with younger age, low education, smoking, exercising, stress, and frequent contact with friends could reduce alcohol-related problems in Korea. The findings of the present study provide information for establishing and promoting country-specific policies and campaigns.

## APPENDIX A. SUPPLEMENTARY DATA

eTable 1. Alcohol consumption according to demographic and behavioral factors, perceived health-related factors, social relations, and past diagnosis of diseases among 43,927 men aged 40–69 in the HEXA-G study

eTable 2. Alcohol consumption according to demographic and behavioral factors, perceived health-related factors, social relations, and past diagnosis of diseases among 85,897 women aged 40–69 in the HEXA-G study

eTable 3. Amount of alcohol consumption based on WHO guideline according to demographic and behavioral factors, perceived health-related factors, social relations, and past diagnosis of diseases among 43,927 men aged 40–69 in the HEXA-G study

eTable 4. Low-, medium-, and high-risk alcohol consumption according to demographic and behavioral factors, perceived health-related factors, social relations, and past diagnosis of diseases among 85,897 women aged 40–69 in the HEXA-G study

eTable 5. Alcohol consumption lower or more than 40 g/day (threshold for low-risk alcohol consumption) according to demographic and behavioral factors, perceived health, social relations, and diagnosis history of diseases among 43,927 men aged 40–69 in the HEXA-G study

eTable 6. Alcohol consumption lower or more than 20 g/day (threshold for low-risk alcohol consumption) according to demographic and behavioral factors, perceived health, social relations, and diagnosis history of diseases among 85,897 women aged 40–69 in the HEXA-G study

eTable 7. Frequency of type of alcoholic beverage consumed according to demographic and behavioral factors, perceived health-related factors, social relations, and past diagnosis of diseases among 32,475 current male drinkers aged 40–69 in the HEXA-G study

eTable 8. Associations of demographic and behavioral factors, perceived health, social relations, and diagnosis history of diseases with the consumption of each type of alcoholic beverage compared to consumption of other types among 32,475 male current drinkers aged 40–69 in the HEXA-G study

eTable 9. Frequency of type of alcoholic beverage consumed according to demographic and behavioral factors, perceived health-related factors, social relations, and past diagnosis of diseases among 26,502 current female drinkers aged 40–69 in the HEXA-G study

eTable 10. Associations of demographic and behavioral factors, perceived health, social relations, and diagnosis history of diseases with the consumption of each type of alcoholic beverage compared to with consumption of other types among 26,502 female current drinkers aged 40–69 in the HEXA-G study
