# Supplementary material for: Trends and Correlates of High-Risk Alcohol Consumption and Types of Alcoholic Beverages in Middle-Aged Korean Adults: Results From the HEXA-G Study
Source: J Epidemiol. 2019 Apr 5;29(4):125–32. doi: 10.2188/jea.JE20170296 (PMC6414807; doi:10.2188/jea.JE20170296)
Supplement: Supplementary file 1 [file je-29-125-s001.pdf]

eTable 1. Alcohol consumption according to demographic and behavioral factors, perceived health-related factors, social relations, and past diagnosis of diseases among 43,927 men aged 40–69 in the HEXA-G study

	Never	Former					Current				
	%	%	cOR	(95% CI)	aOR[a]	(95% CI)	%	cOR	(95% CI)	aOR[a]	(95% CI)
Total, N	8,913	2,539					32,475				
Demographic factors											
Age, years											
40–44	14.2	8.8	1.00	(ref)	1.00	(ref)	20.3	1.00	(ref)	1.00	(ref)
45–49	12.8	9.6	1.22	(1.00–1.49)	1.09	(0.89–1.34)	16.4	0.90	(0.83–0.98)	0.90	(0.82–0.98)
50–54	18.2	17.2	1.53	(1.28–1.83)	1.25	(1.04–1.50)	20.0	0.77	(0.71–0.84)	0.77	(0.71–0.84)
55–59	18.2	20.5	1.82	(1.53–2.17)	1.32	(1.10–1.58)	17.7	0.68	(0.63–0.74)	0.71	(0.65–0.78)
60–64	19.7	23.6	1.93	(1.63–2.29)	1.27	(1.05–1.53)	15.2	0.54	(0.50–0.59)	0.60	(0.55–0.66)
65–69	16.9	20.3	1.95	(1.64–2.32)	1.18	(0.97–1.44)	10.5	0.44	(0.40–0.48)	0.52	(0.47–0.58)
p for trend			<0.001		0.073			<0.001		<0.001	
Education											
≤Middle school	22.9	27.7	1.00	(ref)	1.00	(ref)	20.2	1.00	(ref)	1.00	(ref)
High school	39.2	40.8	0.86	(0.77–0.96)	0.93	(0.82–1.05)	41.1	1.19	(1.12–1.26)	0.95	(0.89–1.01)
≥College	36.4	30.8	0.70	(0.62–0.79)	0.83	(0.73–0.95)	37.5	1.16	(1.09–1.24)	0.94	(0.87–1.01)
p for trend			<0.001		0.009			<0.001		0.099	
Household income, 10,000 won											
<200	29.1	34.5	1.00	(ref)	1.00	(ref)	22.3	1.00	(ref)	1.00	(ref)
200–399.9	39.7	39.0	0.83	(0.75–0.92)	1.05	(0.94–1.18)	43.5	1.43	(1.35–1.52)	1.26	(1.18–1.34)
≥400	21.8	19.4	0.75	(0.66–0.85)	1.05	(0.90–1.22)	27.1	1.62	(1.51–1.73)	1.42	(1.32–1.54)
p for trend			<0.001		0.713			<0.001		<0.001	
Current occupation											
Manual labor	43.9	41.7	1.00	(ref)	1.00	(ref)	47.7	1.00	(ref)	1.00	(ref)
Office	31.3	24.9	0.84	(0.75–0.94)	0.96	(0.85–1.09)	33.9	1.00	(0.94–1.05)	0.98	(0.92–1.04)
Unemployed/house wives	22.3	31.3	1.47	(1.33–1.64)	1.23	(1.09–1.39)	16.2	0.67	(0.63–0.71)	0.88	(0.82–0.94)
Marital status											
Living with spouse	93.6	93.4	1.00	(ref)	1.00	(ref)	94.0	1.00	(ref)	1.00	(ref)
Living alone	5.8	6.4	1.10	(0.92–1.32)	1.15	(0.95–1.40)	5.8	0.99	(0.90–1.09)	0.89	(0.80–0.99)
Behavioral factors											
Smoking status											
Never	46.1	24.0	1.00	(ref)	1.00	(ref)	24.4	1.00	(ref)	1.00	(ref)
Former	31.7	61.4	3.71	(3.34–4.12)	3.38	(3.03–3.76)	40.0	2.38	(2.25–2.51)	2.45	(2.31–2.59)
Current	22.0	14.3	1.25	(1.09–1.44)	1.25	(1.09–1.45)	35.4	3.04	(2.86–3.23)	2.99	(2.81–3.19)
BMI, kg/m^2											
<18.5	1.9	1.8	0.98	(0.70–1.37)	0.96	(0.68–1.36)	1.1	0.61	(0.51–0.73)	0.68	(0.56–0.83)
18.5–24.9	60.0	57.9	1.00	(ref)	1.00	(ref)	58.5	1.00	(ref)	1.00	(ref)
25.0–29.9	35.5	37.1	1.09	(0.99–1.19)	1.05	(0.95–1.16)	37.6	1.09	(1.03–1.14)	1.03	(0.98–1.08)
≥30.0	2.5	3.2	1.30	(1.00–1.69)	1.23	(0.94–1.61)	2.7	1.11	(0.96–1.29)	0.98	(0.84–1.14)
p for trend			0.018		0.109			<0.001		0.062	

Duration of regular exercise, min/week											
None	46.0	41.9	1.00	(ref)	1.00	(ref)	41.9	1.00	(ref)	1.00	(ref)
<150	11.4	11.7	1.12	(0.97–1.30)	1.20	(1.03–1.39)	12.5	1.21	(1.12–1.30)	1.26	(1.17–1.37)
≥150	38.7	43.9	1.25	(1.13–1.37)	1.12	(1.01–1.24)	41.8	1.18	(1.13–1.25)	1.34	(1.27–1.41)
p for trend			<0.001		0.026			<0.001		<0.001	
Perceived health-related factors											
Self-rated health											
Good	45.4	37.9	0.86	(0.77–0.94)	0.92	(0.83–1.02)	46.6	1.00	(0.95–1.05)	1.03	(0.98–1.09)
Normal	40.4	39.4	1.00	(ref)	1.00	(ref)	41.5	1.00	(ref)	1.00	(ref)
Poor	13.6	22.4	1.69	(1.50–1.91)	1.38	(1.21–1.57)	11.4	0.82	(0.76–0.88)	0.85	(0.78–0.92)
p for trend			<0.001		<0.001			<0.001		<0.001	
Perceived stress											
Not at all	63.8	61.2	1.00	(ref)	1.00	(ref)	62.4	1.00	(ref)	1.00	(ref)
Often	29.8	31.5	1.10	(1.00–1.22)	1.06	(0.96–1.17)	31.2	1.07	(1.02–1.13)	1.07	(1.02–1.13)
Frequent	5.3	6.3	1.25	(1.03–1.50)	1.02	(0.84–1.25)	5.6	1.08	(0.98–1.20)	1.15	(1.03–1.29)
p for trend			0.005		0.394			0.006		0.001	
Social relationship											
Contact frequency with family, times/month											
None	6.8	7.3	1.00	(ref)	1.00	(ref)	6.1	1.00	(ref)	1.00	(ref)
<8	36.6	35.6	0.91	(0.76–1.09)	0.96	(0.79–1.17)	38.9	1.18	(1.07–1.30)	0.99	(0.89–1.11)
≥8	54.7	55.2	0.94	(0.79–1.12)	0.92	(0.76–1.12)	53.3	1.08	(0.98–1.19)	0.92	(0.83–1.02)
p for trend			0.971		0.313			0.233		0.007	
Contact frequency with close friends, times/month											
None	10.4	11.9	1.00	(ref)	1.00	(ref)	6.9	1.00	(ref)	1.00	(ref)
<4	40.4	39.0	0.84	(0.73–0.98)	0.97	(0.83–1.14)	42.9	1.61	(1.48–1.75)	1.46	(1.33–1.60)
≥4	47.1	47.2	0.88	(0.76–1.01)	0.97	(0.83–1.13)	48.2	1.55	(1.43–1.69)	1.45	(1.33–1.59)
p for trend			0.308		0.674			<0.001		<0.001	
Past diagnosis of disease											
Diabetes (yes)	9.7	15.6	1.70	(1.50–1.94)	1.29	(1.12–1.48)	8.5	0.86	(0.79–0.93)	0.96	(0.89–1.05)
Myocardial infarction (yes)	4.9	7.5	1.58	(1.32–1.89)	1.15	(0.96–1.39)	3.0	0.60	(0.53–0.67)	0.69	(0.61–0.78)
Stroke (yes)	1.7	5.4	3.33	(2.64–4.22)	2.46	(1.92–3.14)	1.2	0.73	(0.61–0.88)	0.91	(0.75–1.10)
Cancer (yes)	2.7	9.8	3.88	(3.24–4.66)	2.97	(2.45–3.60)	1.5	0.54	(0.46–0.63)	0.62	(0.53–0.73)
Acute liver disease (yes)	0.8	1.4	1.84	(1.23–2.77)	1.61	(1.06–2.45)	0.9	1.10	(0.85–1.44)	1.08	(0.82–1.42)
Fatty liver (yes)	6.3	9.6	1.59	(1.36–1.86)	1.36	(1.15–1.60)	9.5	1.56	(1.42–1.71)	1.56	(1.41–1.72)
Cirrhosis (yes)	3.3	8.2	2.59	(2.16–3.11)	2.34	(1.93–2.84)	2.2	0.64	(0.56–0.74)	0.63	(0.55–0.73)

aOR, adjusted odds ratio; CI, confidence interval; cOR, crude odds ratio.

The total percentage of each category does not equal 100%, because there were missing data.

[a]Odds ratios and confidence intervals were evaluated by multinomial logistic regression adjusted with all variables in the table.

eTable 2. Alcohol consumption according to demographic and behavioral factors, perceived health-related factors, social relations, and past diagnosis of diseases among 85,897 women aged 40–69 in the HEXA-G study

	Never	Former					Current				
	%	%	cOR	(95% CI)	aOR[a]	(95% CI)	%	cOR	(95% CI)	aOR[a]	(95% CI)
Total, N	58,484	911					26,502				
Demographic factors											
Age, years											
40–44	14.5	24.1	1.00	(ref)	1.00	(ref)	27.7	1.00	(ref)	1.00	(ref)
45–49	16.9	16.2	0.58	(0.47–0.72)	0.51	(0.41–0.63)	23.3	0.73	(0.69–0.76)	0.68	(0.65–0.71)
50–54	23.1	22.1	0.57	(0.47–0.70)	0.42	(0.34–0.52)	24.4	0.56	(0.53–0.58)	0.50	(0.48–0.53)
55–59	19.7	18.0	0.55	(0.45–0.67)	0.35	(0.28–0.44)	13.8	0.37	(0.35–0.39)	0.34	(0.32–0.36)
60–64	15.8	12.2	0.46	(0.37–0.58)	0.26	(0.20–0.34)	7.6	0.25	(0.24–0.27)	0.24	(0.22–0.25)
65–69	9.9	7.4	0.45	(0.34–0.59)	0.22	(0.16–0.30)	3.2	0.17	(0.16–0.19)	0.17	(0.15–0.18)
p for trend			<0.001		<0.001			<0.001		<0.001	
Education											
≤Middle school	38.4	45.7	1.00	(ref)	1.00	(ref)	31.9	1.00	(ref)	1.00	(ref)
High school	41.2	35.2	0.72	(0.62–0.83)	0.60	(0.50–0.71)	46.7	1.36	(1.32–1.41)	0.86	(0.83–0.89)
≥College	19.1	18.6	0.82	(0.68–0.98)	0.55	(0.44–0.69)	20.4	1.29	(1.24–1.34)	0.70	(0.67–0.74)
p for trend			0.002		<0.001			<0.001		<0.001	
Household income, 10,000 won											
<200	31.5	41.3	1.00	(ref)	1.00	(ref)	26.3	1.00	(ref)	1.00	(ref)
200–399.9	37.4	33.3	0.68	(0.58–0.79)	0.81	(0.68–0.96)	41.2	1.32	(1.28–1.37)	1.04	(1.00–1.09)
≥400	19.9	18.8	0.72	(0.60–0.86)	0.89	(0.72–1.11)	24.0	1.45	(1.39–1.51)	1.08	(1.03–1.14)
p for trend			<0.001		0.066			<0.001		0.002	
Current occupation											
Manual labor	23.0	25.9	1.00	(ref)	1.00	(ref)	32.2	1.00	(ref)	1.00	(ref)
Office	12.1	14.6	1.07	(0.86–1.33)	1.11	(0.87–1.41)	15.7	0.93	(0.88–0.97)	0.82	(0.77–0.86)
Unemployed/house wives	62.8	56.8	0.80	(0.69–0.94)	0.92	(0.78–1.09)	50.3	0.57	(0.55–0.59)	0.68	(0.65–0.70)
Marital status											
Living with spouse	87.0	74.5	1.00	(ref)	1.00	(ref)	85.5	1.00	(ref)	1.00	(ref)
Living alone	12.7	25.4	2.33	(2.00–2.71)	1.99	(1.69–2.34)	14.3	1.14	(1.10–1.19)	1.29	(1.23–1.35)
Behavioral factors											
Smoking status											
Never	98.0	86.9	1.00	(ref)	1.00	(ref)	93.4	1.00	(ref)	1.00	(ref)
Former	0.6	8.1	14.96	(11.54–19.40)	11.23	(8.57–14.71)	1.8	3.15	(2.75–3.61)	3.04	(2.64–3.51)
Current	1.1	4.7	4.69	(3.42–6.44)	3.33	(2.41–4.61)	4.4	4.11	(3.73–4.52)	3.68	(3.33–4.07)
BMI, kg/m^2											
<18.5	2.1	2.0	0.98	(0.61–1.58)	0.80	(0.50–1.30)	1.8	0.82	(0.74–0.91)	0.72	(0.64–0.80)
18.5–24.9	68.4	64.4	1.00	(ref)	1.00	(ref)	71.7	1.00	(ref)	1.00	(ref)
25.0–29.9	26.4	28.6	1.15	(1.00–1.34)	1.15	(0.99–1.34)	23.9	0.87	(0.84–0.90)	1.01	(0.98–1.05)
≥30.0	3.0	4.9	1.77	(1.30–2.40)	1.42	(1.04–1.95)	2.5	0.82	(0.75–0.89)	0.94	(0.85–1.03)
p for trend			0.001		0.004			<0.001		0.080	

Duration of regular exercise, min/week											
None	49.8	50.2	1.00	(ref)	1.00	(ref)	47.1	1.00	(ref)	1.00	(ref)
<150	11.3	13.5	1.19	(0.97–1.45)	1.38	(1.13–1.69)	12.2	1.14	(1.09–1.20)	1.22	(1.16–1.28)
≥150	35.6	34.7	0.97	(0.84–1.12)	1.21	(1.04–1.41)	37.3	1.11	(1.07–1.14)	1.31	(1.26–1.36)
p for trend			0.740		0.009			<0.001		<0.001	
Perceived health-related factors											
Self-rated health											
Good	34.7	26.6	0.82	(0.70–0.96)	0.88	(0.75–1.04)	38.2	1.08	(1.05–1.12)	1.07	(1.03–1.11)
Normal	45.0	42.0	1.00	(ref)	1.00	(ref)	45.9	1.00	(ref)	1.00	(ref)
Poor	19.5	31.0	1.70	(1.45–1.98)	1.40	(1.19–1.65)	15.3	0.77	(0.74–0.80)	0.86	(0.82–0.90)
p for trend			<0.001		<0.001			<0.001		<0.001	
Perceived stress											
Not at all	52.4	45.8	1.00	(ref)	1.00	(ref)	50.3	1.00	(ref)	1.00	(ref)
Often	37.4	38.4	1.18	(1.02–1.36)	1.03	(0.89–1.19)	39.5	1.10	(1.07–1.14)	1.15	(1.12–1.19)
Frequent	9.2	14.8	1.85	(1.52–2.25)	1.31	(1.06–1.62)	9.4	1.07	(1.02–1.13)	1.20	(1.13–1.27)
p for trend			<0.001		0.035			<0.001		<0.001	
Social relationship											
Contact frequency with family, times/month											
None	4.9	9.2	1.00	(ref)	1.00	(ref)	4.9	1.00	(ref)	1.00	(ref)
<8	35.7	35.1	0.52	(0.41–0.67)	0.66	(0.51–0.85)	36.8	1.04	(0.97–1.11)	0.97	(0.90–1.04)
≥8	57.8	54.2	0.50	(0.40–0.63)	0.67	(0.52–0.86)	57.1	1.00	(0.93–1.07)	0.97	(0.90–1.04)
p for trend			<0.001		0.053			0.075		0.691	
Contact frequency with close friends, times/month											
None	7.8	11.4	1.00	(ref)	1.00	(ref)	5.8	1.00	(ref)	1.00	(ref)
<4	30.0	32.3	0.74	(0.59–0.92)	0.95	(0.75–1.22)	31.5	1.42	(1.33–1.51)	1.27	(1.18–1.36)
≥4	59.7	54.1	0.62	(0.50–0.77)	0.80	(0.64–1.01)	60.4	1.36	(1.28–1.45)	1.25	(1.17–1.33)
p for trend			<0.001		0.009			<0.001		<0.001	
Past diagnosis of disease											
Diabetes (yes)	6.1	7.5	1.25	(0.98–1.61)	1.10	(0.85–1.43)	2.9	0.46	(0.42–0.50)	0.64	(0.59–0.70)
Myocardial infarction (yes)	2.2	4.0	1.85	(1.32–2.59)	1.69	(1.19–2.40)	1.1	0.51	(0.45–0.58)	0.84	(0.73–0.96)
Stroke (yes)	0.9	2.1	2.37	(1.49–3.76)	1.95	(1.21–3.16)	0.4	0.48	(0.39–0.59)	0.74	(0.60–0.92)
Cancer (yes)	4.2	10.6	2.70	(2.18–3.34)	2.75	(2.21–3.43)	2.4	0.55	(0.51–0.60)	0.63	(0.58–0.69)
Acute liver disease (yes)	0.6	1.4	2.54	(1.46–4.44)	2.14	(1.19–3.85)	0.4	0.71	(0.57–0.88)	0.79	(0.63–0.99)
Fatty liver (yes)	3.5	6.0	1.77	(1.34–2.33)	1.54	(1.15–2.05)	3.2	0.90	(0.83–0.98)	1.15	(1.06–1.26)
Cirrhosis (yes)	1.8	3.6	2.04	(1.43–2.90)	1.70	(1.18–2.44)	1.2	0.66	(0.58–0.75)	0.68	(0.59–0.77)

aOR, adjusted odds ratio; CI, confidence interval; cOR, crude odds ratio.

The total percentage of each category does not equal 100%, because there were missing data.

[a]Odds ratios and confidence intervals were evaluated by multinomial logistic regression adjusted with all variables in the table.

eTable 3. Amount of alcohol consumption based on WHO guideline according to demographic and behavioral factors, perceived health-related factors, social relations, and past diagnosis of diseases among 43,927 men aged 40–69 in the HEXA-G study

	Never	Current											
		<40 g/day			40–60 g/day			≥60 g/day					
	%	%	aOR[a]	(95% CI)	%	aOR[a]	(95% CI)	%	aOR[a]	(95% CI)	*p*[b]	*p*[c]	*p*[d]
Total, N	8,913	27,213			2,422			1,999					
Demographic factors													
Age, years													
40–44	14.2	20.3	1.00	(ref)	20.9	1.00	(ref)	19.7	1.00	(ref)			
45–49	12.8	16.2	0.89	(0.81–0.98)	17.4	0.89	(0.76–1.04)	17.4	0.92	(0.78–1.09)	0.999	0.655	0.728
50–54	18.2	19.6	0.77	(0.70–0.84)	21.8	0.74	(0.64–0.86)	22.1	0.78	(0.66–0.91)	0.639	0.871	0.649
55–59	18.2	17.6	0.72	(0.66–0.78)	18.8	0.66	(0.56–0.77)	18.2	0.66	(0.56–0.79)	0.253	0.318	0.984
60–64	19.7	15.5	0.62	(0.57–0.68)	12.1	0.42	(0.35–0.50)	14.4	0.53	(0.44–0.65)	<0.001	0.086	0.045
65–69	16.9	10.8	0.54	(0.49–0.60)	9.1	0.40	(0.33–0.49)	8.3	0.40	(0.32–0.50)	0.002	0.005	0.929
p for trend			<0.001			<0.001			<0.001				
Education													
≤Middle school	22.9	19.3	1.00	(ref)	25.8	1.00	(ref)	26.3	1.00	(ref)			
High school	39.2	40.7	0.98	(0.92–1.05)	43.5	0.75	(0.67–0.85)	44.6	0.80	(0.71–0.92)	<0.001	0.001	0.405
≥College	36.4	38.9	1.00	(0.93–1.08)	29.7	0.58	(0.50–0.67)	28.3	0.61	(0.52–0.71)	<0.001	<0.001	0.649
p for trend			0.028			<0.001			<0.001				
Household income , 10,000 won													
<200	29.1	21.8	1.00	(ref)	24.5	1.00	(ref)	26.7	1.00	(ref)			
200–399.9	39.7	44.2	1.29	(1.20–1.37)	41.8	1.18	(1.04–1.33)	39.7	1.03	(0.90–1.18)	0.132	0.001	0.117
≥400	21.8	27.3	1.43	(1.32–1.55)	26.3	1.52	(1.30–1.76)	26.7	1.45	(1.23–1.70)	0.407	0.874	0.637
p for trend			<0.001			<0.001			<0.001				
Current occupation													
Manual labor	43.9	46.7	1.00	(ref)	53.0	1.00	(ref)	56.2	1.00	(ref)			
Office	31.3	34.7	0.99	(0.93–1.05)	29.6	0.92	(0.82–1.03)	27.7	0.81	(0.72–0.93)	0.171	0.001	0.126
Unemployed/house wives	22.3	16.5	0.89	(0.83–0.96)	15.1	0.88	(0.76–1.01)	14.2	0.73	(0.62–0.85)	0.799	0.008	0.059
Marital status													
Living with spouse	93.6	94.3	1.00	(ref)	92.8	1.00	(ref)	92.3	1.00	(ref)			
Living alone	5.8	5.5	0.86	(0.78–0.96)	7.1	0.94	(0.78–1.14)	7.4	0.96	(0.79–1.17)	0.314	0.884	0.262
Behavioral factors													
Smoking status													
Never	46.1	25.8	1.00	(ref)	15.7	1.00	(ref)	15.1	1.00	(ref)			
Former	31.7	40.0	2.32	(2.19–2.46)	40.3	3.76	(3.31–4.28)	39.8	3.81	(3.30–4.39)	<0.001	<0.001	0.891
Current	22.0	34.1	2.76	(2.59–2.94)	43.9	5.29	(4.64–6.04)	45.1	5.61	(4.86–6.49)	<0.001	<0.001	0.513
BMI, kg/m^2													
<18.5	1.9	1.1	0.67	(0.55–0.82)	1.1	0.67	(0.44–1.03)	1.4	0.86	(0.57–1.31)	0.990	0.216	0.366
18.5–24.9	60.0	59.4	1.00	(ref)	54.7	1.00	(ref)	51.5	1.00	(ref)			
25.0–29.9	35.5	36.8	1.00	(0.95–1.05)	40.7	1.20	(1.09–1.32)	43.4	1.33	(1.20–1.48)	<0.001	<0.001	0.085
≥30.0	2.5	2.6	0.94	(0.80–1.10)	3.6	1.29	(0.99–1.68)	3.7	1.36	(1.03–1.80)	0.008	0.004	0.766
p for trend			0.625			<0.001			<0.001				
Duration of regular exercise, min/week													
None	46.0	41.3	1.00	(ref)	46.7	1.00	(ref)	46.9	1.00	(ref)			
<150	11.4	12.9	1.29	(1.19–1.40)	9.4	0.94	(0.80–1.10)	9.3	0.94	(0.79–1.12)	<0.001	<0.001	0.987
≥150	38.7	42.2	1.33	(1.26–1.41)	40.8	1.34	(1.21–1.48)	41.0	1.35	(1.21–1.51)	0.953	0.813	0.888
p for trend			<0.001			<0.001			<0.001				

Perceived health-related factors													
Self-rated health													
Good	45.4	47.5	1.04	(0.99–1.10)	42.3	0.95	(0.86–1.05)	43.2	1.10	(0.98–1.23)	0.041	0.302	0.026
Normal	40.4	41.3	1.00	(ref)	44.1	1.00	(ref)	39.3	1.00	(ref)			
Poor	13.6	10.7	0.82	(0.76–0.89)	13.1	0.83	(0.71–0.96)	17.1	1.17	(1.01–1.37)	0.939	<0.001	<0.001
p for trend			<0.001			0.428			0.832				
Perceived stress													
Not at all	63.8	63.6	1.00	(ref)	57.4	1.00	(ref)	55.2	1.00	(ref)			
Often	29.8	30.6	1.05	(ref)	33.6	1.21	(ref)	35.0	1.30	(ref)	0.002	<0.001	0.294
Frequent	5.3	5.2	1.08	(0.96–1.21)	8.1	1.62	(1.35–1.96)	8.7	1.64	(1.35–2.00)	<0.001	<0.001	0.936
p for trend			0.047			<0.001			<0.001				
Social relationship													
Contact frequency with family, times/month													
None	6.8	5.6	1.00	(ref)	7.3	1.00	(ref)	9.8	1.00	(ref)			
<8	36.6	38.9	1.05	(0.94–1.17)	40.2	0.96	(0.79–1.17)	36.0	0.65	(0.53–0.79)	0.332	<0.001	0.001
≥8	54.7	54.1	0.99	(0.89–1.11)	51.5	0.80	(0.66–0.97)	53.2	0.60	(0.50–0.72)	0.018	<0.001	0.013
p for trend			0.221			0.001			<0.001				
Contact frequency with close friends, times/month													
None	10.4	6.6	1.00	(ref)	7.6	1.00	(ref)	7.6	1.00	(ref)			
<4	40.4	44.1	1.51	(1.37–1.66)	39.6	1.37	(1.13–1.65)	34.4	1.40	(1.14–1.73)	0.275	0.479	0.831
≥4	47.1	47.6	1.44	(1.32–1.59)	51.4	1.57	(1.31–1.88)	57.0	1.99	(1.63–2.43)	0.341	0.001	0.054
p for trend			<0.001			<0.001			<0.001				
Past diagnosis of disease													
Diabetes (yes)	9.7	8.3	0.96	(0.88–1.04)	8.8	0.98	(0.83–1.15)	9.7	1.00	(0.84–1.19)	0.767	0.544	0.804
Myocardial infarction (yes)	4.9	3.0	0.71	(0.63–0.80)	2.4	0.53	(0.40–0.71)	3.2	0.69	(0.52–0.90)	0.046	0.808	0.186
Stroke (yes)	1.7	1.3	0.94	(0.77–1.14)	1.0	0.70	(0.45–1.09)	1.0	0.66	(0.41–1.06)	0.176	0.132	0.839
Cancer (yes)	2.7	1.6	0.65	(0.55–0.76)	1.1	0.46	(0.31–0.70)	1.1	0.47	(0.30–0.73)	0.100	0.146	0.961
Acute liver disease (yes)	0.8	0.9	1.09	(0.83–1.44)	1.1	1.36	(0.86–2.16)	0.7	0.81	(0.45–1.46)	0.287	0.286	0.119
Fatty liver (yes)	6.3	8.8	1.46	(1.32–1.61)	11.8	1.94	(1.66–2.26)	14.3	2.32	(1.98–2.72)	<0.001	<0.001	0.050
Cirrhosis (yes)	3.3	2.1	0.63	(0.54–0.73)	2.2	0.61	(0.46–0.83)	2.2	0.58	(0.41–0.80)	0.857	0.579	0.760

aOR, adjusted odds ratio; CI, confidence interval.

The total percentage of each category does not equal 100%, because there were missing data.

[a]Odds ratios and confidence intervals were evaluated by multinomial logistic regression adjusted with all variables in the table.

[b]*p* for difference between low-risk and medium-risk alcohol consumption group calculated by testing linear hypotheses about the regression coefficients

[c]*p* for difference between low-risk and high-risk alcohol consumption group calculated by testing linear hypotheses about the regression coefficients

[d]*p* for difference between medium-risk and high-risk alcohol consumption group calculated by testing linear hypotheses about the regression coefficients

eTable 4. Low-, medium-, and high-risk alcohol consumption according to demographic and behavioral factors, perceived health-related factors, social relations, and past diagnosis of diseases among 85,897 women aged 40–69 in the HEXA-G study

	Never	Current											
		<20 g/day			20–40 g/day			≥40 g/day					
	%	%	aOR[1]	(95% CI)	%	aOR[a]	(95% CI)	%	aOR[a]	(95% CI)	*p*[b]	*p*[c]	*p*[d]
Total, N	58,484	24,389			974			408					
Demographic factors													
Age, years													
40–44	14.5	27.6	1.00	(ref)	30.0	1.00	(ref)	26.7	1.00	(ref)			
45–49	16.9	23.0	0.68	(0.65–0.71)	27.2	0.67	(0.56–0.80)	27.7	0.72	(0.55–0.95)	0.902	0.642	0.640
50–54	23.1	24.4	0.51	(0.49–0.53)	24.1	0.38	(0.32–0.46)	27.0	0.44	(0.33–0.58)	0.003	0.290	0.455
55–59	19.7	13.9	0.34	(0.33–0.36)	12.1	0.23	(0.18–0.29)	12.7	0.24	(0.17–0.35)	0.001	0.060	0.743
60–64	15.8	7.8	0.25	(0.23–0.26)	4.6	0.11	(0.08–0.15)	4.9	0.12	(0.07–0.20)	<0.001	0.005	0.743
65–69	9.9	3.4	0.18	(0.16–0.19)	2.0	0.07	(0.05–0.12)	1.0	0.04	(0.01–0.11)	0.001	0.004	0.283
p for trend			<0.001			<0.001			<0.001				
Education													
≤Middle school	38.4	31.6	1.00	(ref)	34.9	1.00	(ref)	38.2	1.00	(ref)			
High school	41.2	46.5	0.86	(0.83–0.90)	50.3	0.77	(0.66–0.90)	48.8	0.72	(0.57–0.92)	0.160	0.139	0.651
≥College	19.1	21.0	0.72	(0.68–0.76)	14.0	0.42	(0.33–0.54)	11.5	0.35	(0.24–0.52)	<0.001	<0.001	0.436
p for trend			<0.001			<0.001			<0.001				
Household income, 10,000 won													
<200	31.5	26.0	1.00	(ref)	31.8	1.00	(ref)	37.3	1.00	(ref)			
200–399.9	37.4	41.5	1.05	(1.00–1.09)	38.6	0.94	(0.79–1.11)	38.2	0.83	(0.65–1.07)	0.214	0.073	0.427
≥400	19.9	24.4	1.09	(1.04–1.15)	22.1	1.06	(0.86–1.30)	15.0	0.64	(0.45–0.89)	0.782	0.002	0.010
p for trend			0.001			0.496			0.018				
Current occupation													
Manual labor	23.0	31.5	1.00	(ref)	43.5	1.00	(ref)	50.0	1.00	(ref)			
Office	12.1	15.9	0.83	(0.79–0.88)	12.9	0.61	(0.49–0.76)	13.0	0.60	(0.43–0.85)	0.007	0.065	0.973
Unemployed/house wives	62.8	50.8	0.69	(0.67–0.72)	42.2	0.50	(0.43–0.58)	35.8	0.35	(0.28–0.44)	<0.001	<0.001	0.009
Marital status													
Living with spouse	87.0	86.2	1.00	(ref)	77.7	1.00	(ref)	73.3	1.00	(ref)			
Living alone	12.7	13.6	1.25	(1.19–1.31)	22.0	1.74	(1.47–2.06)	26.7	2.06	(1.62–2.63)	<0.001	0.249	<0.001
Behavioral factors													
Smoking status													
Never	98.0	94.5	1.00	(ref)	77.1	1.00	(ref)	73.8	1.00	(ref)			
Former	0.6	1.6	2.63	(2.27–3.06)	6.1	11.58	(8.62–15.54)	5.4	9.62	(6.08–15.21)	<0.001	<0.001	0.482
Current	1.1	3.6	3.06	(2.75–3.41)	16.7	14.36	(11.78–17.51)	20.6	17.02	(12.97–22.35)	<0.001	<0.001	0.286
BMI, kg/m^2													
<18.5	2.1	1.9	0.74	(0.67–0.83)	1.3	0.46	(0.26–0.81)	0.5	0.19	(0.05–0.77)	0.101	0.055	0.242
18.5–24.9	68.4	71.8	1.00	(ref)	70.7	1.00	(ref)	66.9	1.00	(ref)			
25.0–29.9	26.4	23.7	1.00	(0.97–1.04)	25.1	1.08	(0.93–1.26)	27.9	1.22	(0.97–1.53)	0.342	0.095	0.383
≥30.0	3.0	2.5	0.92	(0.83–1.01)	2.9	1.02	(0.69–1.51)	4.7	1.61	(1.00–2.61)	0.602	0.023	0.140
p for trend			0.292			0.076			0.001				
Duration of regular exercise, min/week													
None	49.8	47.1	1.00	(ref)	51.4	1.00	(ref)	48.5	1.00	(ref)			
<150	11.3	12.2	1.21	(1.15–1.27)	8.8	0.93	(0.73–1.17)	10.3	1.21	(0.86–1.70)	0.025	0.988	0.201
≥150	35.6	37.5	1.30	(1.26–1.35)	36.6	1.40	(1.21–1.62)	38.2	1.75	(1.40–2.18)	0.327	0.010	0.098
p for trend			<0.001			<0.001			<0.001				

Perceived health-related factors													
Self-rated health													
Good	34.7	38.5	1.07	(1.03–1.11)	39.8	1.30	(1.13–1.51)	33.6	1.13	(0.90–1.43)	0.008	0.649	0.297
Normal	45.0	46.0	1.00	(ref)	42.3	1.00	(ref)	41.4	1.00	(ref)			
Poor	19.5	15.0	0.84	(0.81–0.88)	17.7	0.89	(0.73–1.07)	24.5	1.21	(0.93–1.58)	0.616	0.008	0.056
p for trend			<0.001			<0.001			0.817				
Perceived stress													
Not at all	52.4	50.9	1.00	(ref)	44.3	1.00	(ref)	41.4	1.00	(ref)			
Often	37.4	39.3	1.14	(1.10–1.18)	40.5	1.34	(1.16–1.54)	39.0	1.26	(1.01–1.58)	0.029	0.381	0.656
Frequent	9.2	9.0	1.16	(1.09–1.23)	15.0	1.88	(1.53–2.32)	18.6	2.00	(1.48–2.70)	<0.001	<0.001	0.745
p for trend			<0.001			<0.001			<0.001				
Social relationship													
Contact frequency with family, times/month													
None	4.9	4.6	1.00	(ref)	8.0	1.00	(ref)	8.6	1.00	(ref)			
<8	35.7	36.6	1.00	(0.92–1.08)	32.6	0.66	(0.51–0.87)	31.6	0.67	(0.45–1.00)	0.003	0.052	0.958
≥8	57.8	57.8	1.01	(0.94–1.10)	58.8	0.74	(0.57–0.95)	59.3	0.77	(0.52–1.12)	0.016	0.148	0.863
p for trend			0.351			0.555			0.891				
Contact frequency with close friends, times/month													
None	7.8	5.7	1.00	(ref)	6.9	1.00	(ref)	7.1	1.00	(ref)			
<4	30.0	32.1	1.28	(1.20–1.38)	26.3	1.18	(0.88–1.57)	23.0	1.10	(0.70–1.70)	0.562	0.480	0.781
≥4	59.7	60.5	1.24	(1.16–1.33)	66.0	1.48	(1.13–1.94)	67.9	1.56	(1.04–2.35)	0.211	0.270	0.820
p for trend			0.003			<0.001			0.001				
Past diagnosis of disease													
Diabetes (yes)	6.1	2.9	0.65	(0.60–0.71)	2.4	0.52	(0.34–0.80)	2.2	0.37	(0.19–0.73)	0.321	0.102	0.387
Myocardial infarction (yes)	2.2	1.1	0.83	(0.73–0.95)	1.6	1.36	(0.82–2.27)	1.5	1.09	(0.48–2.48)	0.063	0.525	0.646
Stroke (yes)	0.9	0.4	0.74	(0.59–0.91)	0.6	1.07	(0.47–2.45)	0.5	0.79	(0.19–3.22)	0.380	0.927	0.706
Cancer (yes)	4.2	2.5	0.65	(0.59–0.71)	1.0	0.28	(0.15–0.53)	2.2	0.60	(0.31–1.16)	0.010	0.806	0.111
Acute liver disease (yes)	0.6	0.4	0.74	(0.58–0.95)	0.3	0.51	(0.15–1.68)	1.5	2.56	(1.10–5.95)	0.537	0.005	0.027
Fatty liver (yes)	3.5	3.1	1.13	(1.03–1.23)	3.1	1.11	(0.76–1.62)	7.4	2.43	(1.64–3.60)	0.945	<0.001	0.004
Cirrhosis (yes)	1.8	1.2	0.66	(0.57–0.75)	1.6	0.89	(0.54–1.48)	1.2	0.58	(0.24–1.43)	0.242	0.798	0.412

aOR, adjusted odds ratio; CI, confidence interval.

The total percentage of each category does not equal 100%, because there were missing data.

[a]Odds ratios and confidence intervals were evaluated by multinomial logistic regression adjusted with all variables in the table.

[b]*p* for difference between low-risk and medium-risk alcohol consumption group calculated by testing linear hypotheses about the regression coefficients

[c]*p* for difference between low-risk and high-risk alcohol consumption group calculated by testing linear hypotheses about the regression coefficients

[d]*p* for difference between medium-risk and high-risk alcohol consumption group calculated by testing linear hypotheses about the regression coefficients

eTable 5. Alcohol consumption lower or more than 40 g/day (threshold for low-risk alcohol consumption) according to demographic and behavioral factors, perceived health, social relations, and diagnosis history of diseases among 43,927 men aged 40–69 in the HEXA-G study

	Never	Current						
		<40 g/day			≥40 g/day			
	%	%	aOR[a]	(95% CI)	%	aOR[a]	(95% CI)	p^b
Total, N	8,913	27,213			4,421			
Demographic factors								
Age, years								
40–44	14.2	20.3	1.00	(ref)	20.3	1.00	(ref)	
45–49	12.8	16.2	0.88	(0.81–0.97)	17.4	0.90	(0.79–1.02)	0.791
50–54	18.2	19.6	0.76	(0.70–0.83)	21.9	0.75	(0.66–0.85)	0.799
55–59	18.2	17.6	0.72	(0.66–0.79)	18.5	0.66	(0.58–0.76)	0.149
60–64	19.7	15.5	0.64	(0.59–0.71)	13.1	0.49	(0.42–0.56)	<0.001
65–69	16.9	10.8	0.57	(0.52–0.63)	8.8	0.43	(0.36–0.50)	<0.001
p for trend			<0.001			<0.001		
Education								
≤Middle school	22.9	19.3	1.00	(ref)	26.0	1.00	(ref)	
High school	39.2	40.7	0.94	(0.88–1.01)	44.0	0.74	(0.67–0.82)	<0.001
≥College	36.4	38.9	0.91	(0.84–0.99)	29.1	0.53	(0.47–0.60)	<0.001
p for trend			0.028			<0.001		
Household income, 10,000 won								
<200	29.1	21.8	1.00	(ref)	25.5	1.00	(ref)	
200–399.9	39.7	44.2	1.29	(1.20–1.37)	40.9	1.11	(1.00–1.23)	0.001
≥400	21.8	27.3	1.43	(1.32–1.55)	26.5	1.48	(1.31–1.68)	0.497
p for trend			<0.001			<0.001		
Current occupation								
Manual labor	43.9	46.7	1.00	(ref)	54.4	1.00	(ref)	
Office	31.3	34.7	0.95	(0.89–1.01)	28.8	0.83	(0.75–0.91)	0.002
Unemployed/house wives	22.3	16.5	0.92	(0.85–0.99)	14.7	0.83	(0.74–0.94)	0.070
Marital status								
Living with spouse	93.6	94.3	1.00	(ref)	92.6	1.00	(ref)	
Living alone	5.8	5.5	0.91	(0.82–1.02)	7.2	1.01	(0.86–1.18)	0.967
Behavioral factors								
Smoking status								
Never	46.1	25.8	1.00	(ref)	15.4	1.00	(ref)	
Former	31.7	40.0	2.33	(2.20–2.47)	40.1	3.79	(3.43–4.20)	<0.001
Current	22.0	34.1	2.78	(2.61–2.97)	44.4	5.48	(4.93–6.09)	<0.001
BMI, kg/m^2								
<18.5	1.9	1.1	0.68	(0.55–0.83)	1.2	0.77	(0.55–1.06)	0.420
18.5–24.9	60.0	59.4	1.00	(ref)	53.3	1.00	(ref)	
25.0–29.9	35.5	36.8	0.99	(0.94–1.05)	41.9	1.25	(1.15–1.35)	<0.001
≥30.0	2.5	2.6	0.93	(0.79–1.09)	3.6	1.31	(1.05–1.62)	0.000
p for trend			0.625			<0.001		
Duration of regular exercise, min/week								
None	46.0	41.3	1.00	(ref)	46.8	1.00	(ref)	
<150	11.4	12.9	1.28	(1.18–1.39)	9.3	0.92	(0.81–1.05)	<0.001
≥150	38.7	42.2	1.32	(1.24–1.39)	40.9	1.32	(1.22–1.44)	0.894
p for trend			<0.001			<0.001		
Perceived health								
Self-rated health								
Good	45.4	47.5	1.04	(0.99–1.10)	42.7	1.01	(0.93–1.10)	0.404
Normal	40.4	41.3	1.00	(ref)	41.9	1.00	(ref)	
Poor	13.6	10.7	0.83	(0.76–0.90)	14.9	0.98	(0.87–1.10)	0.001
p for trend			<0.001			0.692		

Perceived stress								
Not at all	63.8	63.6	1.00	(ref)	56.4	1.00	(ref)	
Often	29.8	30.6	1.05	(0.99–1.11)	34.2	1.25	(1.15–1.36)	<0.001
Frequent	5.3	5.2	1.08	(0.96–1.21)	8.4	1.63	(1.40–1.91)	<0.001
p for trend			0.048			<0.001		
Social relationship								
Contact frequency with family, times/month								
None	6.8	5.6	1.00	(ref)	8.4	1.00	(ref)	
<8	36.6	38.9	1.05	(0.94–1.17)	38.3	0.79	(0.68–0.93)	<0.001
≥8	54.7	54.1	0.99	(0.89–1.11)	52.3	0.69	(0.60–0.81)	<0.001
p for trend			0.221			<0.001		
Contact frequency with close friends, times/month								
None	10.4	6.6	1.00	(ref)	7.6	1.00	(ref)	
<4	40.4	44.1	1.49	(1.35–1.64)	37.3	1.37	(1.18–1.59)	0.224
≥4	47.1	47.6	1.42	(1.29–1.56)	54.0	1.72	(1.49–1.99)	0.005
p for trend			<0.001			<0.001		
Diagnosis history of disease								
Diabetes (yes)	9.7	8.3	0.96	(0.88–1.05)	9.2	0.99	(0.87–1.13)	0.538
Myocardial infarction (yes)	4.9	3.0	0.71	(0.62–0.80)	2.7	0.60	(0.49–0.75)	0.115
Stroke (yes)	1.7	1.3	0.94	(0.77–1.15)	1.0	0.69	(0.48–0.97)	0.052
Cancer (yes)	2.7	1.6	0.65	(0.55–0.76)	1.1	0.47	(0.34–0.64)	0.034
Acute liver disease (yes)	0.8	0.9	1.08	(0.82–1.43)	0.9	1.10	(0.74–1.64)	0.932
Fatty liver (yes)	6.3	8.8	1.45	(1.31–1.60)	12.9	2.09	(1.84–2.38)	<0.001
Cirrhosis (yes)	3.3	2.1	0.63	(0.55–0.73)	2.2	0.60	(0.47–0.76)	0.623

aOR, adjusted odds ratio; CI, confidence interval

The total percentage of each category does not equal 100%, because there were missing data.

[a]Odds ratios and confidence intervals were evaluated by multinomial logistic regression adjusted with all variables in the table.

[b]*p* for difference between low-risk and high-risk group estimated by testing linear hypotheses about the regression coefficients

eTable 6. Alcohol consumption lower or more than 20 g/day (threshold for low-risk alcohol consumption) according to demographic and behavioral factors, perceived health, social relations, and diagnosis history of diseases among 85,897 women aged 40–69 in the HEXA-G study

	Never	Current						
		<20 g/day			≥20 g/day			
	%	%	aOR[a]	(95% CI)	%	aOR[a]	(95% CI)	p^b
Total, N	58,484	24,389			1,382			
Demographic factors								
Age, years								
40–44	26.6	27.6	1.00	(ref)	29.0	1.00	(ref)	
45–49	30.9	23.0	0.68	(0.65–0.71)	27.4	0.69	(0.59–0.80)	0.881
50–54	42.4	24.4	0.51	(0.49–0.53)	25.0	0.40	(0.34–0.47)	0.003
55–59	43.4	13.9	0.35	(0.33–0.37)	12.3	0.23	(0.19–0.28)	<.0001
60–64	34.8	7.8	0.25	(0.23–0.27)	4.7	0.11	(0.08–0.15)	<.0001
65–69	21.8	3.4	0.18	(0.16–0.19)	1.7	0.06	(0.04–0.10)	<.0001
p for trend			<0.001			<0.001		
Education								
≤Middle school	38.4	31.6	1.00	(ref)	35.9	1.00	(ref)	
High school	41.2	46.5	0.85	(0.82–0.89)	49.9	0.77	(0.67–0.88)	0.126
≥College	19.1	21.0	0.70	(0.66–0.74)	13.2	0.41	(0.33–0.51)	<.0001
p for trend			<0.001			<0.001		
Household income, 10,000 won								
<200	31.5	26.0	1.00	(ref)	33.4	1.00	(ref)	
200–399.9	37.4	41.5	1.05	(1.00–1.09)	38.5	0.91	(0.79–1.04)	0.047
≥400	19.9	24.4	1.09	(1.04–1.15)	20.0	0.92	(0.77–1.10)	0.066
p for trend			0.001			0.497		
Current occupation								
Manual labor	23.0	31.5	1.00	(ref)	45.4	1.00	(ref)	
Office	12.1	15.9	0.82	(0.78–0.87)	13.0	0.61	(0.50–0.74)	0.002
Unemployed/house wives	62.8	50.8	0.69	(0.67–0.72)	40.3	0.45	(0.40–0.51)	<.0001
Marital status								
Living with spouse	87.0	86.2	1.00	(ref)	76.4	1.00	(ref)	
Living alone	12.7	13.6	1.27	(1.21–1.34)	23.4	1.80	(1.56–2.09)	0.307
Behavioral factors								
Smoking status								
Never	98.0	94.5	1.00	(ref)	76.1	1.00	(ref)	
Former	0.6	1.6	2.64	(2.27–3.07)	5.9	10.96	(8.45–14.22)	<0.001
Current	1.1	3.6	3.07	(2.76–3.42)	17.9	15.10	(12.73–17.91)	<0.001
BMI, kg/m^2								
<18.5	2.1	1.9	0.75	(0.67–0.83)	1.1	0.39	(0.23–0.66)	0.015
18.5–24.9	68.4	71.8	1.00	(ref)	69.6	1.00	(ref)	
25.0–29.9	26.4	23.7	1.00	(0.97–1.04)	25.9	1.12	(0.98–1.27)	0.102
≥30.0	3.0	2.5	0.92	(0.83–1.02)	3.4	1.19	(0.88–1.62)	0.104
p for trend			0.293			0.002		
Duration of regular exercise, min/week								
None	49.8	47.1	1.00	(ref)	50.6	1.00	(ref)	
<150	11.3	12.2	1.21	(1.15–1.27)	9.3	1.01	(0.83–1.22)	0.066
≥150	35.6	37.5	1.30	(1.25–1.34)	37.0	1.50	(1.32–1.70)	0.023
p for trend			<0.001			<0.001		
Perceived health								
Self-rated health								
Good	34.7	38.5	1.07	(1.03–1.11)	38.0	1.25	(1.11–1.42)	0.012
Normal	45.0	46.0	1.00	(ref)	42.0	1.00	(ref)	
Poor	19.5	15.0	0.85	(0.81–0.89)	19.7	0.99	(0.84–1.16)	0.067
p for trend			<0.001			0.001		

Perceived stress								
Not at all	52.4	50.9	1.00	(ref)	43.4	1.00	(ref)	
Often	37.4	39.3	1.14	(1.10–1.18)	40.0	1.31	(1.16–1.48)	0.027
Frequent	9.2	9.0	1.16	(1.10–1.23)	16.1	1.92	(1.61–2.28)	<.0001
p for trend			<0.001			<0.001		
Social relationship								
Contact frequency with family, times/month								
None	4.9	4.6	1.00	(ref)	8.2	1.00	(ref)	
<8	35.7	36.6	1.00	(0.92–1.08)	32.3	0.67	(0.53–0.84)	0.001
≥8	57.8	57.8	1.01	(0.94–1.09)	59.0	0.75	(0.60–0.93)	0.007
p for trend			0.351			0.584		
Contact frequency with close friends, times/month								
None	7.8	5.7	1.00	(ref)	6.9	1.00	(ref)	
<4	30.0	32.1	1.28	(1.19–1.38)	25.3	1.16	(0.91–1.49)	0.436
≥4	59.7	60.5	1.24	(1.16–1.33)	66.6	1.51	(1.20–1.90)	0.089
p for trend			0.003			<0.001		
Diagnosis history of disease								
Diabetes (yes)	6.1	2.9	0.65	(0.60–0.71)	2.3	0.47	(0.33–0.68)	0.089
Myocardial infarction (yes)	2.2	1.1	0.83	(0.73–0.95)	1.6	1.27	(0.82–1.98)	0.065
Stroke (yes)	0.9	0.4	0.73	(0.59–0.91)	0.6	0.99	(0.48–2.03)	0.434
Cancer (yes)	4.2	2.5	0.65	(0.59–0.71)	1.4	0.38	(0.24–0.60)	0.022
Acute liver disease (yes)	0.6	1.4	0.74	(0.58–0.94)	0.4	1.12	(0.55–2.27)	0.258
Fatty liver (yes)	3.5	6.0	1.13	(1.03–1.23)	3.1	1.53	(1.16–2.02)	0.033
Cirrhosis (yes)	1.8	3.6	0.66	(0.57–0.75)	1.2	0.79	(0.51–1.24)	0.410

aOR, adjusted odds ratio; CI, confidence interval

The total percentage of each category does not equal 100%, because there were missing data.

[a]Odds ratios and confidence intervals were evaluated by multinomial logistic regression adjusted with all variables in the table.

[b]*p* for difference between low-risk and high-risk group estimated by testing linear hypotheses about the regression coefficient

eTable 7. Frequency of type of alcoholic beverage consumed according to demographic and behavioral factors, perceived health-related factors, social relations, and past diagnosis of diseases among 32,475 current male drinkers aged 40–69 in the HEXA-G study

	Soju	Other than Soju	Beer	Other than beer	Makgeolli	Other than Makgeolli	Strong spirits	Other than strong spirits	Wine	Other than wine
	%	%	%	%	%	%	%	%	%	%
Total, N	29,817	2,621	14,023	18,409	6,256	26,186	2,518	29,926	1,745	30,701
Demographic factors										
Age, years										
40–44	20.6	34.2	25.9	16.0	17.5	20.9	29.8	19.5	26.6	19.9
45–49	16.7	27.0	18.4	14.8	14.9	16.8	21.4	16.0	16.8	16.4
50–54	20.0	38.9	19.7	20.1	20.3	19.9	19.5	20.0	18.9	20.0
55–59	17.6	36.9	16.1	19.0	19.4	17.3	14.1	18.0	16.6	17.8
60–64	14.9	35.0	12.2	17.4	16.8	14.8	9.3	15.7	12.7	15.3
65–69	10.1	28.1	7.7	12.6	11.0	10.3	5.8	10.9	8.4	10.6
Education										
≤Middle school	20.4	18.5	13.9	25.0	20.1	20.3	6.9	21.4	7.8	20.9
High school	41.6	35.6	38.9	42.8	38.3	41.8	30.8	42.0	24.8	42.1
≥College	36.8	44.8	46.5	30.7	40.8	36.7	61.6	35.5	66.7	35.8
Household income, 10,000 won										
<200	22.2	23.5	17.4	26.0	23.1	22.1	10.0	23.3	11.3	22.9
200–399.9	43.9	39.9	43.1	43.9	41.4	44.1	35.4	44.3	33.6	44.1
≥400	26.9	29.0	34.2	21.6	29.3	26.5	50.1	25.1	49.4	25.8
Current occupation										
Manual labor	48.0	44.5	44.0	50.5	46.0	48.1	32.3	49.0	30.4	48.7
Office	33.8	34.4	40.5	28.9	33.3	34.0	55.0	32.1	52.7	32.8
Unemployed / house wives	16.0	18.7	13.3	18.5	18.3	15.7	10.2	16.7	14.2	16.3
Marital status										
Living with spouse	94.0	93.2	93.8	94.0	93.5	94.1	93.6	94.0	93.6	94.0
Living alone	5.7	6.4	5.9	5.6	6.2	5.7	6.1	5.7	6.0	5.8
Behavioral factors										
Smoking status										
Never	23.4	36.2	27.0	22.5	25.5	24.2	23.3	24.5	30.4	24.1
Former	39.9	40.5	40.3	39.7	44.1	39.0	37.0	40.2	44.3	39.7
Current	36.5	23.2	32.5	37.6	30.2	36.7	39.3	35.1	25.0	36.0
BMI, kg/m^2										
<18.5	1.1	1.6	0.9	1.3	1.3	1.1	0.6	1.2	1.0	1.1
18.5–24.9	58.0	64.6	59.0	58.2	60.5	58.1	51.7	59.1	58.2	58.5
25.0–29.9	38.1	32.0	37.3	37.8	36.0	37.9	43.6	37.1	37.8	37.6
≥30.0	2.8	1.8	2.8	2.7	2.2	2.9	4.1	2.6	3.0	2.7

Duration of regular exercise, min/week										
None	42.3	37.8	38.7	44.4	36.4	43.3	34.6	42.6	30.4	42.6
<150	12.5	12.7	14.9	10.7	14.3	12.1	17.6	12.1	20.2	12.1
≥150	41.5	46.0	42.8	41.1	46.3	40.8	42.5	41.8	43.6	41.7
Perceived health-related factors										
Self-rated health										
Good	46.4	48.3	47.5	45.9	45.4	46.9	45.7	46.7	46.8	46.6
Normal	41.7	40.2	41.5	41.6	41.9	41.4	42.6	41.4	42.3	41.5
Poor	11.4	10.9	10.7	11.9	12.2	11.2	11.4	11.4	10.1	11.4
Perceived stress										
Not at all	62.3	63.2	61.6	63.0	60.1	62.9	57.8	62.7	60.5	62.5
Often	31.4	29.7	32.2	30.4	32.7	30.9	34.6	30.9	32.2	31.2
Frequent	5.6	6.1	5.5	5.7	6.3	5.4	6.8	5.5	6.0	5.6
Social relationship										
Contact frequency with family, times/month										
None	6.2	7.1	5.5	6.8	6.3	6.2	6.0	6.3	4.2	6.4
<8	39.6	38.5	41.3	37.8	42.0	38.8	47.2	38.9	46.2	39.1
≥8	54.2	54.5	51.8	55.5	49.9	55.0	45.4	54.9	47.5	54.5
Contact frequency with close friends, times/month										
None	6.9	8.9	5.7	8.0	6.9	7.0	4.2	7.3	4.5	7.2
<4	43.7	45.1	42.7	44.0	42.9	43.7	44.1	43.7	47.5	43.5
≥4	49.5	45.9	49.8	48.0	47.8	49.3	49.8	49.1	44.9	49.4
Personal disease history										
Diabetes (yes)	8.6	7.5	6.9	9.7	7.5	8.7	8.0	8.5	6.8	8.6
MI (yes)	2.9	3.1	2.5	3.3	3.0	2.9	2.1	3.0	3.4	2.9
Stroke (yes)	1.2	1.6	0.9	1.5	1.2	1.3	0.9	1.3	1.3	1.2
Cancer (yes)	1.4	2.7	1.4	1.6	2.0	1.4	1.0	1.5	1.6	1.5
Acute liver disease (yes)	0.8	1.1	1.0	0.7	0.8	0.9	1.3	0.8	1.5	0.8
Fatty liver (yes)	9.5	9.3	10.7	8.6	11.9	8.9	14.5	9.0	13.5	9.2
Cirrhosis (yes)	2.0	3.6	2.4	2.0	2.5	2.1	2.1	2.2	2.6	2.1

Frequency of cheongju were not shown because of low prevalence (<5% in men and women); there were 34 missing in the information on types of alcoholic beverage consumed; the total percentage of each category does not equal 100%, because there were missing data.

eTable 8. Associations of demographic and behavioral factors, perceived health, social relations, and diagnosis history of diseases with the consumption of each type of alcoholic beverage compared to consumption of other types among 32,475 male current drinkers aged 40–69 in the HEXA-G study

	Soju *vs* other than Soju		Beer *vs* other than beer		Makgeolli *vs* other than Makgeolli		Strong spirits *vs* other than strong spirits		Wine *vs* other than wine	
	aOR[1]	(95% CI)	aOR[a]	(95% CI)	aOR[a]	(95% CI)	aOR[a]	(95% CI)	aOR[a]	(95% CI)
Demographic factors										
Age, years										
40–44	1.00	(ref)	1.00	(ref)	1.00	(ref)	1.00	(ref)	1.00	(ref)
45–49	1.02	(0.88–1.18)	0.78	(0.72–0.84)	1.06	(0.96–1.16)	0.93	(0.83–1.06)	0.78	(0.67–0.92)
50–54	0.82	(0.72–0.95)	0.65	(0.61–0.70)	1.23	(1.12–1.35)	0.77	(0.68–0.87)	0.82	(0.70–0.95)
55–59	0.76	(0.66–0.87)	0.60	(0.55–0.65)	1.33	(1.21–1.47)	0.70	(0.61–0.81)	0.88	(0.75–1.03)
60–64	0.68	(0.59–0.80)	0.51	(0.47–0.56)	1.31	(1.18–1.46)	0.64	(0.54–0.76)	0.84	(0.70–1.02)
65–69	0.60	(0.51–0.71)	0.46	(0.41–0.50)	1.20	(1.06–1.36)	0.65	(0.53–0.80)	0.83	(0.67–1.05)
p for trend	<0.001		<0.001		<0.001		<0.001		0.126	
Education										
≤Middle school	1.00	(ref)	1.00	(ref)	1.00	(ref)	1.00	(ref)	1.00	(ref)
High school	0.89	(0.79–1.00)	1.31	(1.23–1.40)	0.99	(0.91–1.07)	1.61	(1.35–1.92)	1.35	(1.10–1.66)
≥College	0.62	(0.54–0.71)	1.80	(1.67–1.95)	1.21	(1.10–1.33)	2.66	(2.22–3.18)	3.09	(2.52–3.80)
p for trend	<0.001		<0.001		<0.001		<0.001		<0.001	
Household income, 10,000 won										
<200	1.00	(ref)	1.00	(ref)	1.00	(ref)	1.00	(ref)	1.00	(ref)
200–399.9	1.12	(1.00–1.26)	1.06	(0.99–1.13)	0.94	(0.87–1.02)	1.25	(1.07–1.46)	1.14	(0.95–1.36)
≥400	1.02	(0.89–1.17)	1.42	(1.32–1.53)	1.06	(0.96–1.16)	2.29	(1.95–2.69)	2.05	(1.70–2.46)
p for trend	0.787		<0.001		0.164		<0.001		<0.001	
Current occupation										
Manual labor	1.00	(ref)	1.00	(ref)	1.00	(ref)	1.00	(ref)	1.00	(ref)
Office	1.10	(0.99–1.22)	1.09	(1.03–1.15)	0.90	(0.84–0.96)	1.45	(1.31–1.61)	1.27	(1.12–1.44)
Unemployed/house wives	1.11	(0.98–1.26)	1.02	(0.94–1.10)	1.07	(0.98–1.17)	1.17	(0.99–1.38)	1.28	(1.07–1.52)
Marital status										
Living with spouse	1.00	(ref)	1.00	(ref)	1.00	(ref)	1.00	(ref)	1.00	(ref)
Living alone	0.77	(0.65–0.91)	1.08	(0.98–1.20)	1.19	(1.06–1.35)	1.20	(1.00–1.44)	1.37	(1.10–1.69)
Behavioral factors										
Smoking status										
Never	1.00	(ref)	1.00	(ref)	1.00	(ref)	1.00	(ref)	1.00	(ref)
Former	1.53	(1.39–1.68)	0.89	(0.84–0.95)	1.07	(0.99–1.14)	1.06	(0.95–1.19)	0.97	(0.86–1.09)
Current	2.29	(2.05–2.55)	0.69	(0.65–0.73)	0.82	(0.76–0.89)	1.29	(1.15–1.44)	0.63	(0.55–0.72)
BMI, kg/m^2										
<18.5	0.72	(0.52–1.00)	0.84	(0.67–1.05)	1.22	(0.95–1.57)	0.67	(0.39–1.16)	1.07	(0.65–1.77)
18.5–24.9	1.00	(ref)	1.00	(ref)	1.00	(ref)	1.00	(ref)	1.00	(ref)
25.0–29.9	1.32	(1.21–1.44)	0.91	(0.86–0.95)	0.89	(0.84–0.94)	1.23	(1.13–1.34)	0.93	(0.84–1.03)
≥30.0	1.70	(1.26–2.30)	0.88	(0.77–1.02)	0.72	(0.59–0.87)	1.49	(1.19–1.86)	0.95	(0.71–1.28)
p for trend	<0.001		0.001		<0.001		<0.001		0.201	

Duration of regular exercise, min/week										
None	1.00	(ref)	1.00	(ref)	1.00	(ref)	1.00	(ref)	1.00	(ref)
<150	0.97	(0.85–1.11)	1.31	(1.22–1.41)	1.35	(1.23–1.47)	1.39	(1.23–1.58)	1.71	(1.48–1.98)
≥150	0.91	(0.83–1.00)	1.11	(1.05–1.17)	1.29	(1.21–1.37)	1.15	(1.04–1.27)	1.19	(1.05–1.34)
p for trend	0.047		<0.001		<0.001		0.009		0.020	
Perceived health										
Self-rated health										
Good	0.99	(0.91–1.08)	1.00	(0.95–1.05)	0.94	(0.88–1.00)	0.95	(0.87–1.05)	0.92	(0.83–1.03)
Normal	1.00	(ref)	1.00	(ref)	1.00	(ref)	1.00	(ref)	1.00	(ref)
Poor	1.01	(0.87–1.16)	0.99	(0.91–1.07)	1.07	(0.98–1.18)	1.01	(0.88–1.17)	0.96	(0.80–1.14)
p for trend	0.757		0.906		0.003		0.279		0.300	
Perceived stress										
Not at all	1.00	(ref)	1.00	(ref)	1.00	(ref)	1.00	(ref)	1.00	(ref)
Often	1.04	(0.95–1.13)	1.09	(1.04–1.15)	1.12	(1.06–1.20)	1.19	(1.09–1.31)	1.09	(0.98–1.22)
Frequent	0.87	(0.73–1.03)	1.09	(0.98–1.21)	1.24	(1.10–1.41)	1.42	(1.19–1.70)	1.31	(1.05–1.62)
p for trend	0.584		0.001		<0.001		<0.001		0.022	
Social relationship										
Contact frequency with family, times/month										
None	1.00	(ref)	1.00	(ref)	1.00	(ref)	1.00	(ref)	1.00	(ref)
<8	1.12	(0.94–1.34)	1.15	(1.03–1.27)	1.09	(0.96–1.23)	1.03	(0.85–1.25)	1.49	(1.15–1.93)
≥8	1.06	(0.89–1.26)	1.00	(0.90–1.11)	0.90	(0.80–1.02)	0.73	(0.60–0.88)	1.20	(0.93–1.55)
p for trend	0.735		<0.001		<0.001		<0.001		0.039	
Contact frequency with close friends, times/month										
None	1.00	(ref)	1.00	(ref)	1.00	(ref)	1.00	(ref)	1.00	(ref)
<4	1.24	(1.05–1.45)	1.01	(0.91–1.12)	0.96	(0.85–1.08)	1.20	(0.96–1.49)	1.10	(0.86–1.42)
≥4	1.40	(1.19–1.64)	1.21	(1.09–1.33)	0.97	(0.86–1.09)	1.43	(1.15–1.77)	1.05	(0.82–1.34)
p for trend	<0.001		<0.001		0.993		<0.001		0.583	
Diagnosis history of disease										
Diabetes (yes)	1.24	(1.06–1.44)	0.82	(0.76–0.90)	0.77	(0.69–0.86)	1.11	(0.94–1.30)	0.84	(0.69–1.03)
Myocardial infarction (yes)	1.04	(0.82–1.31)	0.98	(0.85–1.12)	0.93	(0.79–1.10)	0.86	(0.64–1.15)	1.36	(1.03–1.80)
Stroke (yes)	0.84	(0.61–1.18)	0.77	(0.62–0.96)	0.89	(0.69–1.14)	0.91	(0.59–1.41)	1.21	(0.78–1.88)
Cancer (yes)	0.58	(0.44–0.75)	1.08	(0.90–1.31)	1.26	(1.02–1.55)	0.78	(0.51–1.19)	1.14	(0.76–1.69)
Acute liver disease (yes)	0.78	(0.53–1.17)	1.25	(0.98–1.60)	0.95	(0.70–1.28)	1.34	(0.91–1.96)	1.66	(1.10–2.52)
Fatty liver (yes)	0.99	(0.86–1.14)	1.28	(1.19–1.39)	1.36	(1.24–1.49)	1.50	(1.32–1.70)	1.39	(1.20–1.62)
Cirrhosis (yes)	0.54	(0.43–0.68)	1.22	(1.04–1.42)	1.15	(0.96–1.39)	0.92	(0.69–1.23)	1.21	(0.88–1.65)

aOR, adjusted odds ratio; CI, confidence interval.

Results on cheongju were not shown because of low prevalence (<5% in men and women). The total percentage of each category does not equal 100%, because there were missing data.

[a]Odds ratios and confidence intervals were evaluated by multinomial logistic regression adjusted with all variables in the table.

eTable 9. Frequency of type of alcoholic beverage consumed according to demographic and behavioral factors, perceived health-related factors, social relations, and past diagnosis of diseases among 26,502 current female drinkers aged 40–69 in the HEXA-G study

	Soju	Other than Soju	Beer	Other than beer	Makgeolli	Other than Makgeolli	Strong spirits	Other than strong spirits	Wine	Other than wine
	%	%	%	%	%	%	%	%	%	%
Total, N	18,824	7,644	14,711	11,757	3,925	22,543	748	25,721	2,906	23,569
Demographic factors										
Age, years										
40–44	25.4	42.4	34.0	19.7	25.7	28.0	28.6	27.6	30.7	27.3
45–49	23.6	28.9	24.3	22.1	22.5	23.4	27.8	23.2	22.9	23.3
50–54	25.2	28.7	22.8	26.5	25.1	24.3	25.1	24.4	24.1	24.5
55–59	14.6	55.6	11.3	16.9	14.5	13.7	11.5	13.9	12.5	14.0
60–64	8.0	29.6	5.5	10.1	8.4	7.4	4.7	7.6	6.7	7.7
65–69	3.3	14.8	2.0	4.7	3.8	3.1	2.3	3.3	3.1	3.3
Education										
≤Middle school	35.9	22.0	24.7	41.0	31.1	32.1	17.0	32.4	18.3	33.6
High school	46.5	47.0	49.2	43.5	46.1	46.7	50.3	46.5	41.6	47.3
≥College	16.5	30.1	25.4	14.2	22.2	20.1	32.4	20.1	39.2	18.1
Household income, 10,000 won										
<200	28.4	20.9	22.8	30.6	25.3	26.5	20.3	26.4	17.8	27.3
200–399.9	41.2	41.1	41.5	40.7	39.9	41.4	33.6	41.4	36.1	41.8
≥400	22.0	29.1	28.0	19.0	26.8	23.6	34.4	23.7	36.8	22.4
Current occupation	8.4	8.8	7.6	9.6	8.0	8.6	11.8	8.4	9.4	8.4
Manual labor										
Office	34.0	27.8	30.5	34.4	29.8	32.7	30.5	32.3	19.3	33.8
Unemployed / house wives	14.5	18.6	18.8	11.8	15.9	15.6	21.5	15.5	22.7	14.8
Marital status	49.7	51.8	49.1	51.8	53.1	49.8	46.3	50.4	56.0	49.6
Living with spouse										
Living alone	85.3	86.1	86.0	84.9	86.5	85.3	76.1	85.8	85.0	85.6
Behavioral factors										
Smoking status	14.5	13.7	13.9	14.8	13.3	14.4	23.8	14.0	14.7	14.2
Never										
Former	92.9	94.5	94.0	92.6	94.8	93.1	84.4	93.6	94.9	93.2
Current	1.9	1.8	1.8	1.8	1.9	1.8	4.1	1.8	1.8	1.8
BMI, kg/m^2	4.9	3.2	3.9	5.1	3.0	4.7	10.8	4.3	2.9	4.6
<18.5										
18.5–24.9	1.5	2.7	2.1	1.5	1.6	1.9	2.0	1.8	2.6	1.7
25.0–29.9	70.2	75.2	74.1	68.7	72.8	71.5	77.9	71.5	75.6	71.2
≥30.0	25.5	20.1	21.6	26.7	23.0	24.1	18.6	24.1	20.1	24.4

Duration of regular exercise, min/week										
None	47.8	45.4	45.8	48.7	38.8	48.6	40.2	47.3	39.1	48.1
<150	11.7	13.3	13.3	10.8	14.5	11.8	15.9	12.1	17.8	11.5
≥150	36.8	38.4	37.6	36.8	44.1	36.1	38.8	37.2	39.4	37.0
Perceived health-related factors										
Self-rated health										
Good	38.3	38.0	39.6	36.4	36.7	38.4	35.8	38.2	37.7	38.2
Normal	45.5	46.8	45.7	46.0	46.9	45.7	47.1	45.9	47.1	45.7
Poor	15.7	14.5	14.1	16.9	15.7	15.3	15.6	15.3	14.3	15.5
Perceived stress										
Not at all	50.5	49.6	50.1	50.4	49.5	50.4	40.8	50.5	46.4	50.7
Often	39.3	40.0	40.0	38.9	39.5	39.5	43.0	39.4	44.0	39.0
Frequent	9.4	9.5	9.1	9.8	10.2	9.3	14.7	9.3	8.7	9.5
Social relationship										
Contact frequency with family, times/month										
None	5.0	4.7	4.6	5.3	4.8	4.9	7.0	4.9	4.1	5.0
<8	36.8	38.2	37.7	36.1	39.4	36.7	41.3	37.1	41.3	36.6
≥8	58.2	57.1	56.7	58.7	54.4	58.4	50.3	58.1	53.1	58.4
Contact frequency with close friends, times/month										
None	5.9	6.1	5.2	6.7	6.0	5.9	5.5	5.9	4.8	6.0
<4	31.7	33.6	31.5	32.4	29.0	32.7	29.8	32.3	31.9	32.1
≥4	62.4	60.3	61.3	60.9	62.2	61.4	60.6	61.8	59.7	61.8
Personal disease history										
Diabetes (yes)	3.1	2.2	2.1	3.7	3.1	2.8	1.9	2.9	2.0	3.0
MI (yes)	1.2	0.9	0.9	1.4	1.3	1.1	1.7	1.1	1.2	1.1
Stroke (yes)	0.5	0.4	0.3	0.5	0.3	0.4	0.5	0.4	0.4	0.4
Cancer (yes)	2.2	2.9	2.3	2.5	3.2	2.2	1.3	2.4	2.6	2.3
Acute liver disease (yes)	0.4	0.4	0.4	0.5	0.3	0.4	1.2	0.4	0.6	0.4
Fatty liver (yes)	3.3	2.9	2.9	3.5	3.7	3.1	4.7	3.1	3.8	3.1
Cirrhosis (yes)	1.1	1.5	1.2	1.2	1.7	1.1	1.3	1.2	1.1	1.2

Frequency of cheongju were not shown because of low prevalence (<5% in men and women). There were 34 missing in the information on types of alcoholic beverage consumed. The total percentage of each category does not equal 100%, because there were missing data.

eTable 10. Associations of demographic and behavioral factors, perceived health, social relations, and diagnosis history of diseases with the consumption of each type of alcoholic beverage compared to with consumption of other types among 26,502 female current drinkers aged 40–69 in the HEXA-G study

	Soju *vs* other than Soju		Beer *vs* other than beer		Makgeolli *vs* other than Makgeolli		Strong spirits *vs* other than strong spirits		Wine *vs* other than wine	
	aOR[1]	(95% CI)	aOR[a]	(95% CI)	aOR[a]	(95% CI)	aOR[a]	(95% CI)	aOR[a]	(95% CI)
Total, N										
Demographic factors										
Age, years										
40–44	1.00	(ref)	1.00	(ref)	1.00	(ref)	1.00	(ref)	1.00	(ref)
45–49	1.17	(1.08–1.26)	0.72	(0.66–0.77)	1.04	(0.94–1.15)	1.32	(1.08–1.62)	1.08	(0.97–1.21)
50–54	1.09	(1.01–1.18)	0.62	(0.57–0.66)	1.15	(1.04–1.28)	1.33	(1.07–1.64)	1.32	(1.18–1.48)
55–59	1.08	(0.98–1.19)	0.52	(0.47–0.57)	1.20	(1.06–1.36)	1.26	(0.96–1.66)	1.33	(1.16–1.54)
60–64	1.04	(0.92–1.18)	0.44	(0.39–0.49)	1.32	(1.13–1.53)	1.04	(0.71–1.53)	1.40	(1.16–1.67)
65–69	0.79	(0.67–0.94)	0.36	(0.31–0.42)	1.47	(1.20–1.80)	1.23	(0.72–2.09)	1.63	(1.27–2.10)
p for trend	0.538		<0.001		<0.001		0.171		<0.001	
Education										
≤Middle school	1.00	(ref)	1.00	(ref)	1.00	(ref)	1.00	(ref)	1.00	(ref)
High school	0.63	(0.58–0.68)	1.39	(1.30–1.48)	1.06	(0.97–1.16)	2.13	(1.70–2.66)	1.62	(1.44–1.83)
≥College	0.36	(0.33–0.40)	1.86	(1.70–2.04)	1.20	(1.06–1.35)	3.14	(2.41–4.10)	3.63	(3.16–4.16)
p for trend	<0.001		<0.001		0.004		<0.001		<0.001	
Household income, 10,000 won										
<200	1.00	(ref)	1.00	(ref)	1.00	(ref)	1.00	(ref)	1.00	(ref)
200–399.9	0.88	(0.82–0.95)	1.01	(0.95–1.08)	1.00	(0.91–1.10)	1.06	(0.85–1.32)	1.14	(1.01–1.29)
≥400	0.79	(0.72–0.87)	1.20	(1.11–1.31)	1.14	(1.02–1.27)	1.66	(1.31–2.11)	1.72	(1.51–1.96)
p for trend	<0.001		<0.001		0.026		<0.001		<0.001	
Current occupation										
Manual labor	1.00	(ref)	1.00	(ref)	1.00	(ref)	1.00	(ref)	1.00	(ref)
Office	1.08	(0.99–1.18)	1.10	(1.01–1.20)	1.02	(0.90–1.14)	0.92	(0.73–1.16)	1.37	(1.20–1.56)
Unemployed/house wives	0.87	(0.82–0.93)	1.08	(1.02–1.14)	1.03	(0.95–1.12)	0.91	(0.76–1.09)	1.66	(1.50–1.85)
Marital status										
Living with spouse	1.00	(ref)	1.00	(ref)	1.00	(ref)	1.00	(ref)	1.00	(ref)
Living alone	0.91	(0.84–0.99)	1.16	(1.08–1.25)	0.95	(0.85–1.05)	1.98	(1.63–2.41)	1.35	(1.19–1.52)
Behavioral factors										
Smoking status										
Never	1.00	(ref)	1.00	(ref)	1.00	(ref)	1.00	(ref)	1.00	(ref)
Former	1.12	(0.91–1.37)	0.97	(0.81–1.17)	0.98	(0.76–1.26)	2.18	(1.49–3.20)	0.86	(0.64–1.17)
Current	1.55	(1.34–1.80)	0.73	(0.65–0.83)	0.68	(0.56–0.83)	2.45	(1.90–3.16)	0.67	(0.53–0.85)
BMI, kg/m^2										
<18.5	0.69	(0.57–0.83)	1.03	(0.85–1.25)	0.92	(0.70–1.20)	0.86	(0.51–1.46)	1.24	(0.95–1.60)
18.5–24.9	1.00	(ref)	1.00	(ref)	1.00	(ref)	1.00	(ref)	1.00	(ref)
25.0–29.9	1.19	(1.11–1.28)	0.91	(0.85–0.96)	0.94	(0.86–1.02)	0.83	(0.68–1.00)	0.92	(0.83–1.02)
≥30.0	1.30	(1.08–1.57)	0.80	(0.68–0.94)	1.02	(0.82–1.27)	0.61	(0.33–1.13)	0.74	(0.55–1.00)
p for trend	<0.001		<0.001		0.395		0.033		0.005	

Duration of regular exercise, min/week										
None	1.00	(ref)	1.00	(ref)	1.00	(ref)	1.00	(ref)	1.00	(ref)
<150	0.91	(0.84–1.00)	1.23	(1.13–1.34)	1.49	(1.34–1.66)	1.47	(1.18–1.84)	1.63	(1.45–1.83)
≥150	0.95	(0.89–1.01)	1.08	(1.02–1.15)	1.48	(1.37–1.60)	1.27	(1.07–1.51)	1.16	(1.06–1.28)
p for trend	0.078		0.003		<0.001		0.005		0.001	
Perceived health										
Self-rated health										
Good	1.08	(1.01–1.14)	1.06	(1.00–1.12)	0.91	(0.84–0.98)	0.97	(0.82–1.15)	0.94	(0.86–1.03)
Normal	1.00	(ref)	1.00	(ref)	1.00	(ref)	1.00	(ref)	1.00	(ref)
Poor	1.05	(0.97–1.15)	0.94	(0.87–1.01)	1.00	(0.90–1.11)	0.92	(0.74–1.15)	0.95	(0.84–1.08)
p for trend	0.228		0.003		0.029		0.831		0.524	
Stress										
Not at all	1.00	(ref)	1.00	(ref)	1.00	(ref)	1.00	(ref)	1.00	(ref)
Often	0.94	(0.88–0.99)	1.09	(1.04–1.15)	1.03	(0.96–1.11)	1.34	(1.14–1.57)	1.29	(1.18–1.40)
Frequent	0.86	(0.78–0.96)	1.11	(1.01–1.22)	1.17	(1.04–1.33)	1.95	(1.54–2.48)	1.17	(1.01–1.36)
p for trend	0.005		0.001		0.078		<0.001		<0.001	
Social relationship										
Contact frequency with family, times/month										
None	1.00	(ref)	1.00	(ref)	1.00	(ref)	1.00	(ref)	1.00	(ref)
<8	0.99	(0.86–1.14)	1.05	(0.93–1.19)	1.12	(0.94–1.33)	0.83	(0.60–1.14)	1.19	(0.97–1.47)
≥8	1.01	(0.88–1.16)	0.99	(0.88–1.12)	0.95	(0.80–1.12)	0.67	(0.49–0.91)	0.99	(0.81–1.22)
p for trend	0.597		0.149		<0.001		0.001		0.002	
Contact frequency with close friends, times/month										
None	1.00	(ref)	1.00	(ref)	1.00	(ref)	1.00	(ref)	1.00	(ref)
<4	1.20	(1.05–1.36)	0.97	(0.86–1.09)	0.80	(0.69–0.94)	0.95	(0.66–1.36)	0.92	(0.75–1.12)
≥4	1.26	(1.11–1.42)	1.08	(0.96–1.21)	0.92	(0.79–1.07)	1.07	(0.76–1.51)	0.97	(0.80–1.18)
p for trend	0.001		0.001		0.127		0.229		0.500	
Diagnosis history of disease										
Diabetes (yes)	1.15	(0.96–1.38)	0.79	(0.68–0.92)	1.01	(0.82–1.23)	0.72	(0.42–1.25)	0.77	(0.58–1.02)
Myocardial infarction (yes)	1.17	(0.89–1.54)	0.95	(0.75–1.20)	1.14	(0.83–1.54)	1.88	(1.06–3.36)	1.25	(0.86–1.80)
Stroke (yes)	1.03	(0.67–1.59)	0.93	(0.63–1.36)	0.67	(0.37–1.19)	1.42	(0.51–3.94)	1.10	(0.60–2.05)
Cancer (yes)	0.72	(0.61–0.86)	1.00	(0.85–1.18)	1.35	(1.10–1.65)	0.56	(0.30–1.06)	1.10	(0.86–1.41)
Acute liver disease (yes)	0.89	(0.58–1.35)	0.79	(0.53–1.17)	0.69	(0.37–1.26)	2.91	(1.43–5.92)	1.50	(0.88–2.57)
Fatty liver (yes)	1.00	(0.85–1.17)	1.02	(0.88–1.17)	1.14	(0.95–1.37)	1.46	(1.02–2.10)	1.30	(1.05–1.61)
Cirrhosis (yes)	0.67	(0.53–0.84)	1.01	(0.80–1.27)	1.57	(1.19–2.06)	1.05	(0.55–2.01)	0.89	(0.61–1.30)

aOR, adjusted odds ratio; CI, confidence interval

Results on cheongju were not shown because of low prevalence (<5% in men and women). The total percentage of each category does not equal 100%, because there were missing data.

[a]Odds ratios and confidence intervals were evaluated by multinomial logistic regression adjusted with all variables in the table.
